# Enhancing Multi‐Enzyme Cascade Activity in Metal‐Organic Frameworks via Controlled Enzyme Encapsulation

**DOI:** 10.1002/smll.202503059

**Published:** 2025-04-08

**Authors:** Wenqing Fan, Zefang Yu, Dominique Appadoo, Kang Liang, Jieying Liang

**Affiliations:** ^1^ School of Chemical Engineering Australian Centre for NanoMedicine The University of New South Wales Sydney NSW 2052 Australia; ^2^ Graduate School of Biomedical Engineering The University of New South Wales Sydney NSW 2052 Australia; ^3^ Australian Synchrotron‐ANSTO Clayton VIC 3168 Australia

**Keywords:** biocatalysis, incompatible cascades, metal‐organic frameworks, multi‐enzymes, nanobiohybrids

## Abstract

To position multi‐enzymes in a core‐shell structure, the conventional layer‐by‐layer approach is often used. However, this method is time‐consuming and complex, requiring multiple steps and the isolation of intermediates at each stage. To address this challenge, a sequential strategy is introduced for the controlled encapsulation of multi‐enzymes within metal‐organic frameworks (MOFs), achieving a core‐shell structure without the need for intermediate isolation. Synchrotron Terahertz‐Far‐Infrared (THz‐Far‐IR) spectroscopy is employed to monitor this encapsulation process. The results revealed that the first enzyme is co‐precipitated within the MOFs, followed by biomineralization upon the addition of a second enzyme, achieving distinct enzyme positioning. This approach is applicable to both two‐enzyme and three‐enzyme cascade systems. The results demonstrate that multi‐enzyme cascade activity is significantly enhanced compared to conventional one‐pot and layer‐by‐layer approaches, owing to optimal spatial arrangement, increased surface area, and improved enzyme conformation. Furthermore, the encapsulated enzymes exhibit strong resistance to high temperatures, proteolysis, and organic solvents, along with excellent reusability, making this method highly promising for industrial biocatalytic applications.

## Graphical abstract

A sequential strategy is developed for controlled encapsulation of multi‐enzymes within a core‐shell structure in metal‐organic frameworks. The results demonstrate that multi‐enzyme cascade activity is significantly enhanced due to the optimal spatial arrangement, increased surface area, and improved enzymes conformation. Additionally, the encapsulated enzymes exhibit strong resistance to harsh environments and excellent reusability, making them promising for industrial applications.

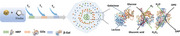

## 1 Introduction

Multi‐enzyme cascade reactions, wherein multiple enzymes work sequentially to catalyze a series of biochemical transformations,^[^ ^1^ ^]^ are essential in nature and have significant applications in industrial biocatalysis,^[^ ^2^ ^]^ medicine and chemical synthesis.^[^ ^3^ ^]^ These reactions offer advantages such as substrate channeling, reduced by‐product formation, and enhanced reaction efficiency.^[^ ^4^ ^]^ However, the direct use of free enzymes in cascade systems often suffers from issues such as poor enzyme stability, low reusability, and enzyme incompatibility,^[^ ^5^ ^]^ which can diminish their practical applicability. Metal‐organic frameworks (MOFs) constructed by coordinating metal ions with organic linkers have emerged as a promising platform to overcome these limitations.^[^ ^6^ ^]^ MOFs provide a highly tunable porous environment that can encapsulate and protect enzymes while maintaining their activity and facilitating cascade reactions through confined spaces.^[^ ^7^ ^]^

There are many strategies have been developed to immobilise enzymes in MOFs,^[^ ^8^ ^]^ including surface adsorption, covalent bonding, post‐filtration, co‐precipitation, and biomineralization. Co‐precipitation and biomineralization are two key strategies used to immobilize enzymes within MOFs. Co‐precipitation refers to the simultaneous deposition of different components,^[^ ^9^ ^]^ such as metal ions, organic linkers, and enzymes,^[^ ^10^ ^]^ leading to the formation of a biocomposite material. This approach provides the benefit of allowing enzyme encapsulation without considering the enzyme size, charge, or other inherent properties, thereby streamlining the synthesis process.^[^ ^11^ ^]^ Furthermore, co‐precipitation minimizes enzyme leaching and improves loading efficiency, particularly for larger biomolecules.^[^ ^9^ ^]^ However, the enzymes participate directly in the nucleation and growth of the framework, which is a biomineralization process.^[^ ^12^ ^]^ Traditional methods for immobilization of multi‐enzyme often involve co‐precipitation or biomineralization of enzymes within a single MOF, but the random positioning of enzymes reduces their cascade efficiency.^[^ ^13^ ^]^ Multi‐enzymes also can be post‐filtrated within MOFs, which helps preserve enzymatic activity and structural integrity by spatially isolating enzymes and minimizing aggregation.^[^ ^14^ ^]^ However, this method is limited to enzymes smaller than the MOF pore size, as pore mismatching can lead to low loading efficiency or high leaching rates.^[^ ^15^ ^]^ To construct core‐shell structures, layer‐by‐layer assembly method has been developed.^[^ ^16^ ^]^ This technique involves the gradual, controlled overgrowth of each shell layer onto the previous one through epitaxial alignment, allowing for the precise encapsulation of multiple enzymes within multi‐shelled MOFs.^[^ ^16c^ ^]^ However, this method is time‐consuming and complex, requiring multiple steps and the isolation of intermediates at each stage.^[^ ^17^ ^]^

In recent years, the one‐pot strategy has gained significant attention for simplifying the synthesis of multicomponent systems. This approach involves introducing multiple components into a single reactor, enabling multistep reactions to proceed without the need for intermediate isolation or changes in reaction conditions.^[^ ^18^ ^]^ To simplify core‐shell enzyme encapsulation in MOFs, we explored the possibility of a sequential strategy that eliminates intermediate isolation at each stage. Although all steps occur within the same reactor, the process may still involve distinct reaction stages.^[^ ^19^ ^]^ To date, few studies have successfully incorporated multiple enzymes into a core‐shell MOF framework within a single reaction system without intermediate isolation. Conventional methods often require polymer modification to alter enzyme surface charges, adding complexity to the process.^[^ ^20^ ^]^ Moreover, due to the multi‐component nature of the sequential reaction, understanding the reaction mechanism and enzyme encapsulation process is essential. Synchrotron Terahertz‐Far‐Infrared (THz‐Far‐IR) spectroscopy, is a powerful analytical technique that utilizes synchrotron radiation to probe materials in the terahertz and far‐infrared regions of the electromagnetic spectrum; it provides insights into molecular vibrations, low‐energy lattice modes, and interactions within complex materials.^[^ ^21^ ^]^ It has been commonly used to study bonding interactions, structural dynamics, and phase transitions,^[^ ^22^ ^]^ making it useful for investigating the stepwise formation and interaction mechanisms in sequential reactions.

**Scheme 1:**
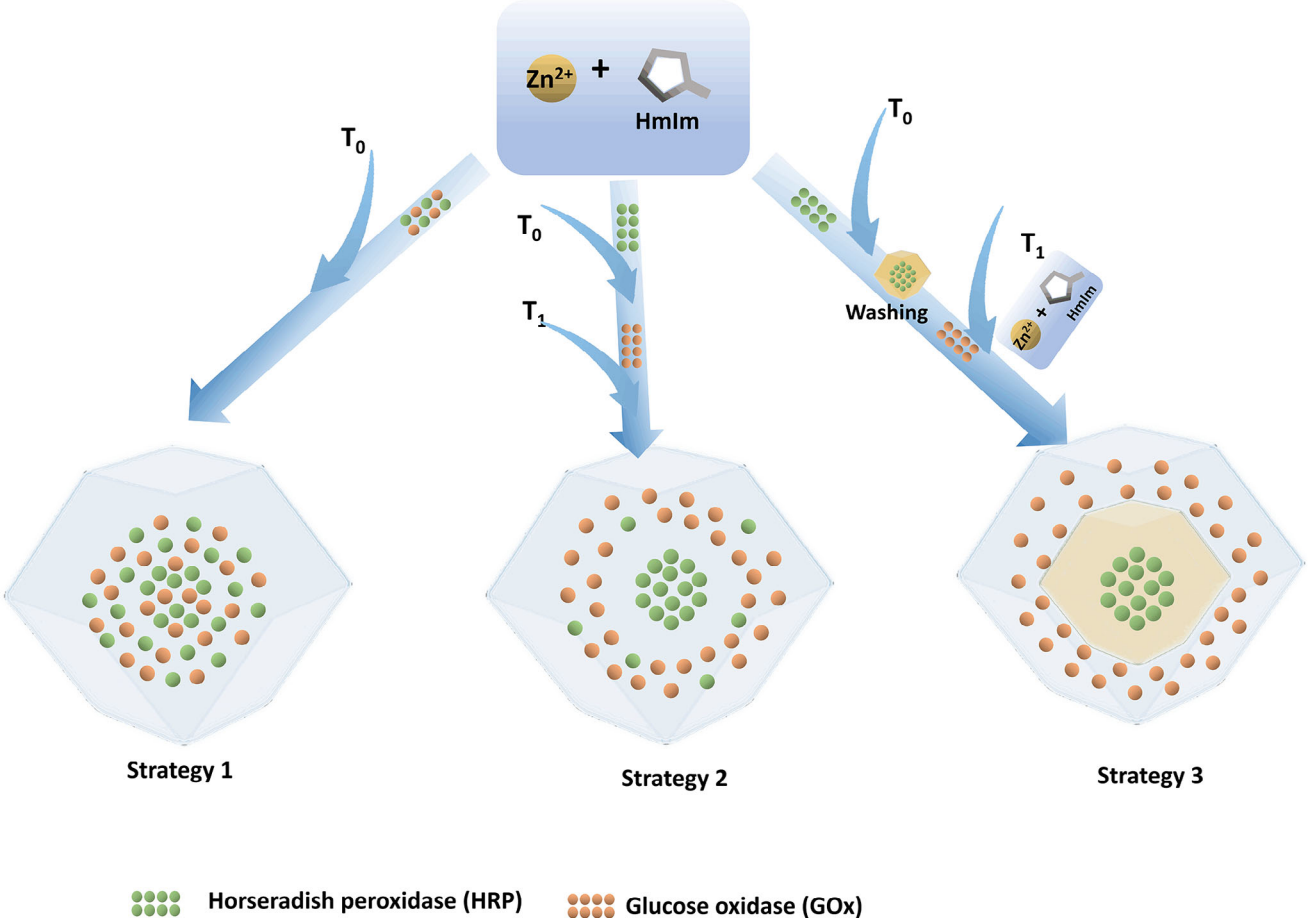
Three strategies were developed for multi‐enzyme encapsulation in MOFs: in Strategy 1, both enzymes are added simultaneously at the start (T_0_) and react for 4 h; in Strategy 2, one enzyme is added at T_0_ to form the core over 2 h, followed by another enzyme at T_1_ (2 h later) to form the shell; in Strategy 3, one enzyme is added at T_0_ and reacts for 2 h, the core is separated, and another enzyme with MOF precursors is added at T_1_ to form the core‐shell structure.

Herein, we present an innovative sequential approach for positioning multiple enzymes within a core‐shell structure by sequentially adding different enzymes during the MOF formation process without the need for intermediate isolation (Strategy 2). This method is compared with the traditional one‐pot approach (Strategy 1) and the layer‐by‐layer strategy (Strategy 3) (**Scheme** **1**). THz‐Far‐IR spectroscopy demonstrated Strategy 2 including a co‐precipitation followed by a biomineralization process, enabling the successful sequential encapsulation of multiple enzymes. This strategy is applicable to both two‐enzyme and three‐enzyme cascade systems, offering significant improvements in multi‐enzyme cascade reactions compared to the traditional one‐pot approach and the layer‐by‐layer strategy. The enhancements arise from the controlled spatial arrangement of enzymes within the core‐shell structure, which optimizes enzyme proximity, minimizes cross‐reactivity, and boosts substrate channeling efficiency between layers. Furthermore, the system benefits from increased surface area and improved enzyme conformation. The resulting core‐shell microreactors also exhibit enhanced stability under high temperatures, proteolysis, and exposure to organic solvents, making them a highly efficient platform for multi‐enzyme biocatalysis in industrial applications.

## 2 Results and Discussion

### 2.1 Synthesis and Characterization of Multi‐Enzyme@ZIF‐8 Composites

Three strategies were used to encapsulate the glucose oxidase (GOx) and horseradish peroxidase (HRP) in ZIF‐8. In Strategy 1, HRP and GOx solutions were simultaneously added with ZIF‐8 precursors for 4 h, and GOx/HRP@ZIF‐8‐1 was obtained. In Strategy 2, HRP or GOx was first encapsulated in ZIF‐8, and after 2 h reaction, another enzyme was introduced, which was denoted as GOx/HRP@ZIF‐8‐2 or HRP/GOx@ZIF‐8‐2. In Strategy 3, after encapsulating the first enzyme (HRP or GOx) for 2 h, the mixture was centrifuged to remove any unbound enzymes and MOF precursors prior to adding the subsequent enzymes and new MOF precursors, which was denoted as GOx/HRP@ZIF‐8‐3 and HRP/GOx@ZIF‐8‐3 (Scheme 1).

**Figure 1:**
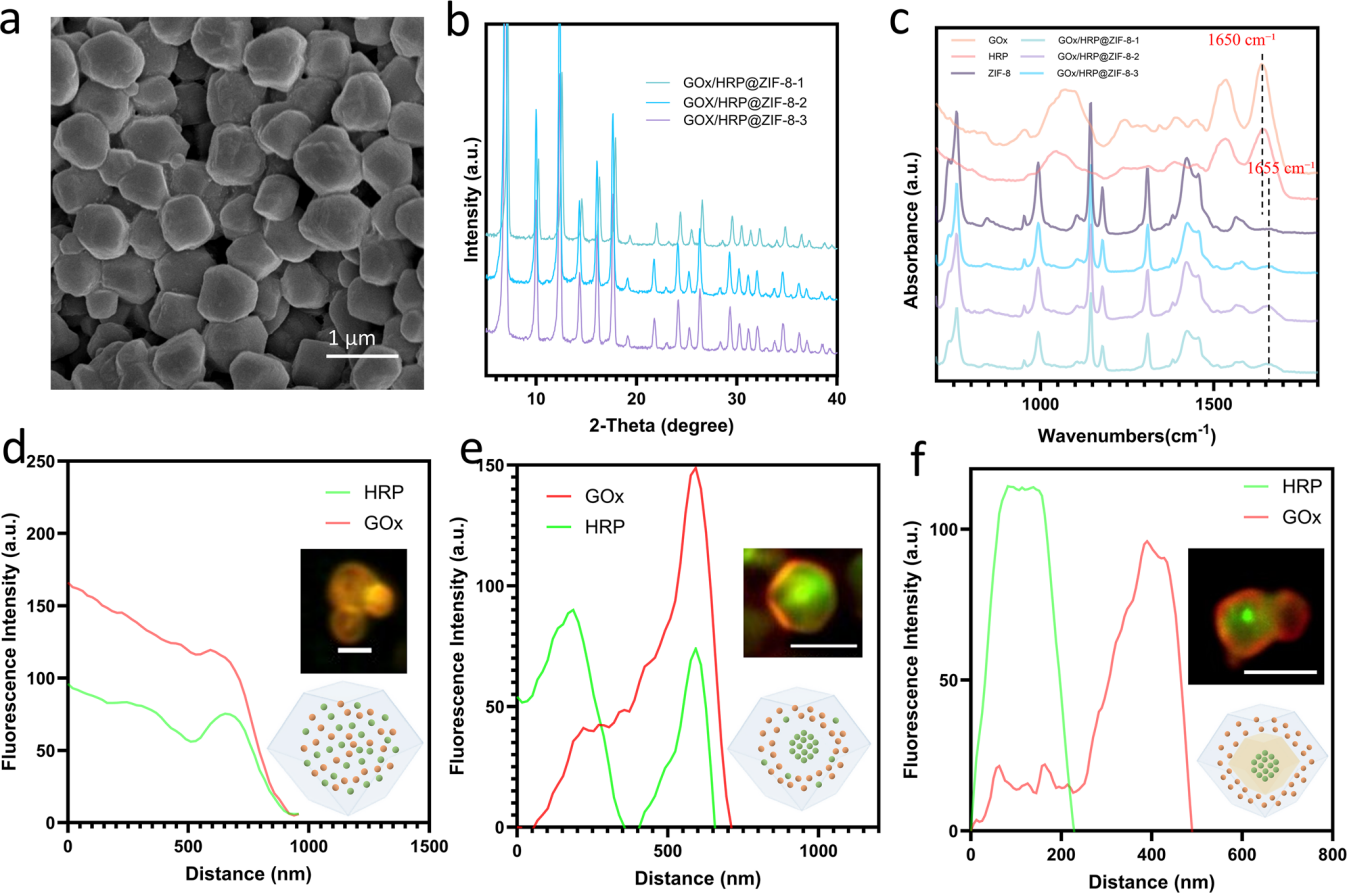
Characterization of the two‐enzyme@MOF samples. a) SEM image of GOx/HRP@ZIF‐8‐2. b) XRD patterns of GOx/HRP@ZIF‐8‐1, GOx/HRP@ZIF‐8‐2, and GOx/HRP@ZIF‐8‐3. c) FTIR spectra of GOx, HRP, ZIF‐8, GOx/HRP@ZIF‐8‐1, GOx/HRP@ZIF‐8‐2, and GOx/HRP@ZIF‐8‐3, with dashed lines indicating the amide I peak of enzymes at 1650 cm⁻¹ for the free enzymes and 1655 cm⁻¹ after encapsulation in MOF. d–f) Fluorescence intensity profiles of MOF samples, calculated based on the inserted fluorescence microscopy images, their schematic images are also inserted. The scale bar is 1 µm. d) GOx/HRP@ZIF‐8‐1, e) GOx/HRP@ZIF‐8‐2, and f) GOx/HRP@ZIF‐8‐3.

Scanning electron microscopy (SEM) images show that all samples exhibit the characteristic dodecahedral morphology typical of ZIF‐8 (**Figure** **1**a; Figure S1, Supporting Information), consistent with the morphology of pure ZIF‐8 (Figure S2, Supporting Information). The X‐ray diffraction (XRD) patterns confirm the crystalline structure of all samples. The peaks for all enzyme‐embedded MOF samples align well with both the simulated and experimentally measured ZIF‐8 patterns (Figure 1b; Figure S3, Supporting Information), indicating that the incorporation of enzymes and the different encapsulation strategies did not disrupt the crystalline framework of ZIF‐8. The Fourier transform infrared (FTIR) spectra (Figure 1c; Figure S4, Supporting Information) show amide I peak which is mainly corresponding to the C═O stretching vibration at 1650 cm is observed slightly shift to 1655 cm⁻¹ in the enzyme‐embedded MOF samples, while this peak is absent in the ZIF‐8 sample without enzymes (Figure 1c), suggesting that the presence of the enzymes is successfully embedded within the MOF structure and the enzymes conformational structure slightly changes upon immobilization. This can be attributed to a combination of hydrophobic interactions,^[^ ^23^ ^]^ electrostatic forces,^[^ ^24^ ^]^ van der Waals interactions,^[^ ^25^ ^]^ and hydrogen bonding,^[^ ^26^ ^]^ among other factors. Furthermore, our previous research demonstrated a symbiotic stability reinforcement effect between the MOF matrix and proteins, driven by coordinative interactions between Zn centers and the functional groups of biomolecules.^[^ ^27^ ^]^ Additionally, the physical constraints imposed by the surrounding pores^[^ ^28^ ^]^ further help to stabilize enzyme conformation within the MOF structure.

Fluorescence microscopy images reveal the spatial positioning of the enzymes, the mixed multi‐enzyme‐ZIF‐8‐1 shows no obvious spatial differentiation, and distinct layers are visible in two‐enzyme‐ZIF‐8‐2 and two‐enzyme‐ZIF‐8‐3. In these samples, GOx was labelled with Atto 633 NHS ester and HRP with Atto 550 NHS ester. Fluorescence colocalization analysis, calculated as fluorescence intensity from the crystal center to the edge radius, was performed using ImageJ to further elucidate the spatial distribution of enzymes within each sample. The GOx/HRP@ZIF‐8‐1 shows no clear distinction between the two enzymes (Figure 1d; Figure S5a, Supporting Information). However, only one enzyme is present in the core, while both enzymes are located in the outer layers with partial overlap in GOx/HRP@ZIF‐8‐2 and HRP/GOx@ZIF‐8‐2 (Figure 1e; Figure S5b,c, Supporting Information). Enzyme loading efficiency demonstrates that 32% HRP was encapsulated in the core, with a final total encapsulation efficiency of 40.5%, suggesting that ≈8.5% of HRP overlaps with the region where GOx is encapsulated (Figure S6, Supporting Information). The fluorescence intensity of enzymes in GOx/HRP@ZIF‐8‐3 and HRP/GOx@ZIF‐8‐3 show distinct peaks, demonstrating the totally separated enzymes position (Figure 1f; Figure S5d,e, Supporting Information).

### 2.2 Synchrotron THz‐Far‐IR Spectroscopy

THz‐Far‐IR spectroscopy was used to analyze the characteristic Zn─N stretching and bending vibrations within the ZIF‐8 framework (Figure S7, Supporting Information). Notably, peaks at ≈168, 278, and 293 cm⁻¹ indicate Zn─N stretching, which are absent in the MOF precursors (Figure S8, Supporting Information). This observation confirms the stability of the metal‐ligand coordination structure, essential for maintaining MOF integrity.^[^ ^21^, ^27^ ^]^ The peak ≈420 cm⁻¹ (Zn─N─C and N─C─Me) indicates ligand bending, associated with in‐plane and out‐of‐plane deformations of aromatic rings.^[^ ^21^, ^22^ ^]^ The consistent appearance and intensity of these peaks across different samples support the successful formation of the ZIF‐8 crystal structure in each strategy (Figure S9, Supporting Information).

**Figure 2:**
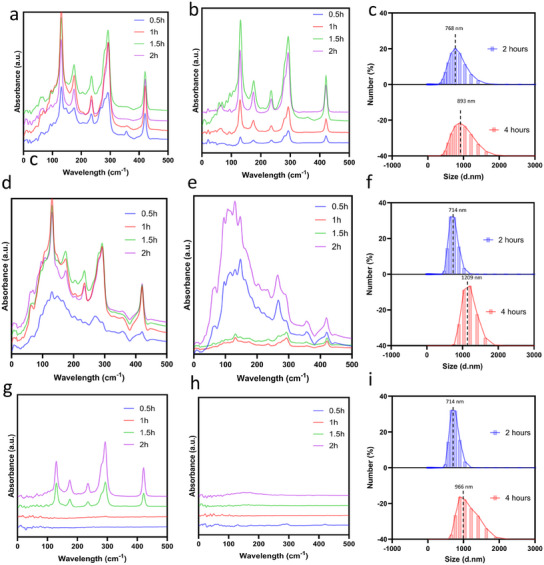
Synchrotron THz‐Far‐IR spectra for tracking sequential core‐shell encapsulation of multi‐enzyme in MOF. a,b) In Strategy 1, synthesis with (a) or without (b) enzyme addition in the prior 2 h. c) Particle size analysis of GOx/HRP@ZIF‐8‐1 at 2 and 4 h of synthesis via DLS. d,e) In post 2 h phase of Strategy 2, where pre‐formed MOFs and free enzymes were removed by filtration using Amicon® Ultra‐0.5 centrifugal filter tube (cut off 30 KDa), with (d) or without (e) second enzyme addition for another 2 h reaction. f) Particle size analysis of GOx/HRP@ZIF‐8‐2 via DLS. g,h) In Strategy 3, a dilution MOF precursors (2.27 mm Zn(NO_3_)·6H_2_O and 43.09 mm HmIm) with (g) or without (h) second enzyme addition. i) Particle size analysis of GOx/HRP@ZIF‐8‐3 via DLS.

To further track this process, MOF formation at various time points was investigated during the synthesis of ZIF‐8 and GOx/HRP@ZIF‐8‐2, with samples taken at 30 min intervals up to 2 h. The spectra reveal that both MOFs formed within 0.5 h with the addition of 45.38 mm Zn(NO_3_)·6H_2_O and 861.75 mm HmIm with or without enzyme addition (**Figure** **2**a,b), indicating it is a coprecipitation process. Dynamic light scattering (DLS) analysis reveals that the particle size of GOx/HRP@ZIF‐8‐1 increased from 560 nm at 0.5 h to 893 nm after 4 h, indicating the gradual formation of the MOF (Figure 2c; Figure S10, Supporting Information). To track the progress of Strategy 2 during the post‐2 h phase, the reaction mixture was filtered by filtration using Amicon Ultra‐0.5 centrifugal filter tube (cut off 30 KDa) to remove pre‐formed MOFs and enzymes. The concentrations of HmIm and Zn after 2 h of reaction were determined using ¹H nuclear magnetic resonance and inductively coupled plasma analysis, respectively (Figure S11, Supporting Information). The concentrations of HmIm and Zn(NO₃)₂·6H₂O were measured to be 476.9 mM and 1.42 mm, respectively. The addition of a second enzyme resulted in the appearance of characteristic MOF peaks after 1 h, confirming MOF formation (Figure 2d). However, in the absence of the second enzyme, these MOF peaks were not prominent (Figure 2e), indicating that the second stage of GOx/HRP@ZIF‐8‐2 formation after 2 h involves a biomineralization process. Furthermore, when pre‐formed MOFs were centrifuged, the second enzyme were subsequently added, MOF formation occurred within 1 h (Figure S12, Supporting Information). This is consistent with the behavior of the filtered samples after the prior 2 h reaction, indicating that MOF formation was not independent but enzyme‐promoted, further supporting the biomineralization mechanism. The particle size GOx/HRP@ZIF‐8‐2 increased from 714 nm at 2 h to 1200 nm at 4 h (Figure 2f), suggesting the MOF shell grew around the core, but not from the additional MOF. In Strategy 3, control experiments were conducted by synthesizing MOFs with and without enzyme addition in a diluted MOF precursors (2.27 mm Zn(NO_3_)·6H_2_O and 43.09 mm HmIm). Results showed that MOF formation occurred only in the presence of enzymes (Figure 2g), and no MOFs formed without them (Figure 2h), suggesting it is a biomineralization process. The particle size of GOx/HRP@ZIF‐8‐3 increased from 714 nm at 2 h to 966 nm at 4 h (Figure 2i), also suggesting the MOF shell grew around the core, but not form the additional MOFs.

The above results indicate that, while spontaneous nucleation can occur at higher MOF precursors concentration via coprecipitation, whereas enzymes are essential to facilitate nucleation via biomineralization at lower precursor concentrations. The second stages in both Strategy 2 and Strategy 3 involve biomineralization, suggesting that it is not necessary to remove the MOF precursors and enzymes to construct the core‐shell structure, which is easy operation and time‐saving. Hence, Strategy 2 is the most direct and efficient method for synthesizing a core‐shell structure of multi‐enzyme in MOFs.

### 2.3 Performance of Two‐Enzyme Cascade Reaction in MOFs

**Figure 3:**
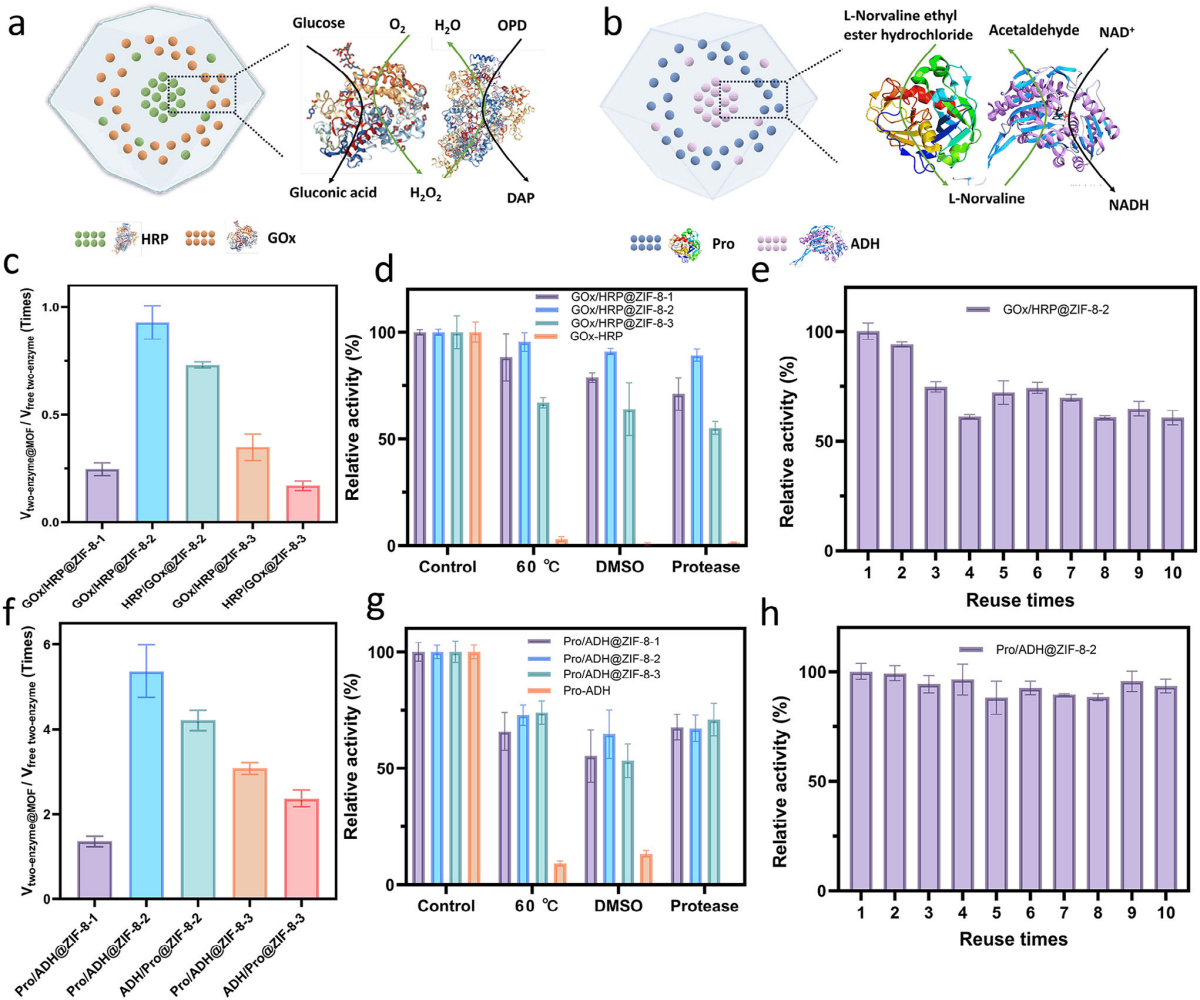
Catalytic performance and stability of the two‐enzyme@MOF samples. a) Reaction mechanism of GOx and HRP. b) Reaction mechanism of Pro and ADH. c) The relative activity (V_enzymes‐MOF_ and V_free enzyme_) of the initial reaction rates (V) between two‐enzyme@MOF samples and free two‐enzyme for GOx‐HRP model. d) The relative activity of GOx/HRP@ZIF‐8‐1, GOx/HRP@ZIF‐8‐2, GOx/HRP@ZIF‐8‐3, and free GOx‐HRP under adverse environments. e) The reusability test of GOx/HRP@ZIF‐8‐2 in ten consecutive cycles. f) The relative activity (V_enzymes‐MOF_ and V_free enzyme_) of the initial reaction rates (V) between two‐enzyme@MOF samples and free two‐enzyme for Pro‐ADH model. g) The relative activity of Pro/ADH@ZIF‐8‐1, Pro/ADH@ZIF‐8‐2, Pro/ADH@ZIF‐8‐3, and free Pro‐ADH under adverse environments. h) The reusability of Pro/ADH@ZIF‐8‐2 in ten consecutive cycles.

To evaluate the cascade activity of GOx‐HRP, glucose was used as the substrate with o‐phenylenediamine (OPD) serving as the indicator.^[^ ^13^, ^16^ ^]^ The reaction was induced by GOx‐catalyzed oxidation of glucose to produce hydrogen peroxide, HRP was then used to oxidize OPD, forming 2,3‐diaminophenazine (DAP) (**Figure** **3**a). The encapsulation efficiency of GOx and HRP in the MOFs was calculated using fluorescent spectrophotometry based on standard curves for each enzyme (Table S1). The results show that the relative activity of GOx/HRP@ZIF‐8‐1 retains 25% of the activity of its free enzyme counterparts. However, HRP/GOx@ZIF‐8‐2 and GOx/HRP@ZIF‐8‐2 retains 73% and 93% of the activity of its free enzyme counterparts at the same enzymes’ concentration, respectively. Therefore, the enzyme cascade activity follows the order: GOx/HRP@ZIF‐8‐2 > HRP/GOx@ZIF‐8‐2 > GOx/HRP@ZIF‐8‐1 (Figure 3c; Figure S13 and Table S2, Supporting Information). In Strategy 3, HRP/GOx@ZIF‐8‐3 and GOx/HRP@ZIF‐8‐3 show only 17% and 35% of the activity of its free enzyme counterparts. The activity of GOx/HRP@ZIF‐8‐2 and GOx/HRP@ZIF‐8‐3, with GOx positioned in the outer layer, is higher than that of HRP/GOx@ZIF‐8‐2 and HRP/GOx@ZIF‐8‐3, where HRP is located in the inner layer. This suggests that positioning GOx in the outer layer, where it can more readily access the substrate, enhances catalytic efficiency.^[^ ^13^, ^20^ ^]^

To exclude the potential effect of MOF precursor concentration on the post‐2 h reaction between HRP/GOx@ZIF‐8‐2 and HRP/GOx@ZIF‐8‐3, identical HRP/GOx@ZIF‐8‐2 MOF precursors, synthesized for 2 h as shown in Figure S11 (Supporting Information), were added to GOx/HRP@ZIF‐8‐3 after 2 h of synthesis. The results demonstrated that HRP/GOx@ZIF‐8‐2 exhibited twice the cascade activity of GOx/HRP@ZIF‐8‐3. Therefore, the improved catalytic performance of GOx/HRP@ZIF‐8‐2 cannot be attributed to differences in precursor concentration (Figure S14, Supporting Information). Moreover, sodium dodecyl sulfate (SDS) treatment was applied to HRP‐GOx‐adsorbed ZIF‐8 and GOx/HRP@ZIF‐8‐2 samples. After SDS washing, the enzyme activity of HRP‐GOx‐adsorbed ZIF‐8 was significantly reduced, retaining only 26.8% of its initial activity. In contrast, GOx/HRP@ZIF‐8‐2 maintained 90.6% of its enzyme activity (Figure S15, Supporting Information), indicating most enzymes are encapsulated inside the MOFs. An additional experiment was conducted to explore the impact of enzymes positioning within the MOFs. Two variations were synthesized: HRP@MOF, where HRP was added at the start of synthesis to form an inner‐core encapsulation, and MOF@HRP, where HRP was introduced after 2 h reaction to create an outer‐layer encapsulation. The activity of MOF@HRP was 14% higher than HRP@MOF (Figure S16, Supporting Information). To examine the tertiary enzyme structure of MOF@HRP and HRP@MOF, the deconvoluted attenuated total reflectance‐Fourier transform infrared (ATR‐FTIR) spectra show five peaks of enzymes, which correspond to α‐helix, β‐sheet, intermolecular β‐sheet, β‐turn, and random coil structures.^[^ ^20^ ^]^ Quantitative analysis reveals that (Figure S17, Supporting Information) the content of α‐helix, β‐sheet in MOF@HRP and HRP@MOF is consistent, whereas β‐turn and intermolecular β‐sheet peaks are slightly lower in MOF@HRP, indicating less structural tightness and altered interaction forces due to biomineralization encapsulation process.^[^ ^20^ ^]^ Nitrogen adsorption analysis showed that the GOx/HRP@ZIF‐8‐2 exhibited a higher surface area (1919.55 m^2^ g^−1^) compared to GOx/HRP@ZIF‐8‐1 (1374.66 m^2^ g^−1^), indicating greater porosity and surface accessibility of GOx/HRP@ZIF‐8‐2 that synthesized by the co‐precipitation followed by biomineralization process than those synthesized by the co‐precipitation process (Figure S18 and Table S3, Supporting Information). Therefore, optimal multi‐enzyme spatial arrangement, increased surface area, as well as the optimized enzyme conformation in Strategy 2 leads to a more effective cascade activity.

The stability of the enzymes‐MOF composites under adverse conditions was further evaluated (Figure 3d). The samples were incubated at 60 °C for 1 h, exposed to dimethyl sulfoxide (DMSO) for 1 h, or treated with proteases for 2 h. After exposure, free enzymes lost all activity, while the MOF‐encapsulated samples retained over 60% of their initial activity. The reusability of the enzymes‐MOF composites was assessed. GOx/HRP@ZIF‐8‐2 retained over 60% of its activity after ten consecutive cycles (Figure 3e), while more than 50% of GOx/HRP@ZIF‐8‐1 and GOx/HRP@ZIF‐8‐3 activity was maintained after five reuse cycles (Figure S19, Supporting Information), demonstrating the considerable operational stability of the enzyme‐MOF composites.

Incompatible multi‐enzyme reactions have been widely used in pharmaceutical synthesis,^[^ ^16^, ^29^ ^]^ biofuel production,^[^ ^30^ ^]^ and complex metabolic engineering pathways.^[^ ^31^ ^]^ In this system, enzymes interfere with each other's function or react unfavorably in the same environment. This incompatibility can lead to enzyme degradation, reduced activity, or undesirable side reactions, making it challenging to conduct multi‐enzyme cascade reactions in a single system.^[^ ^32^ ^]^ Therefore, it is necessary to compartment different enzymes in an isolated interspace with specific circumstances. Strategy 2 can also be extended to incompatible enzymes, such as protease (Pro) and alcohol dehydrogenase (ADH), where Pro not only reacts with its substrate but also digest ADH, potentially reducing ADH activity. ADH and Pro were encapsulated in MOF via Strategy 1, 2, and 3. SEM, XRD, and THz‐Far‐IR results confirmed the ZIF‐8 crystal structure (Figures S20–S22, Supporting Information), and FTIR spectra show amide I peak which is mainly corresponding to the C═O stretching vibration shifts from 1650 to 1655 cm^−1^ upon immobilization (Figure S23, Supporting Information). Fluorescence imaging was conducted using Atto 633 NHS ester labeled ADH and Atto 550 NHS ester labeled Pro. In Pro/ADH@ZIF‐8‐1, the enzymes are homogeneously distributed with no clear spatial separation. In Pro/ADH@ZIF‐8‐2 and ADH/Pro@ZIF‐8‐2, partial colocalization is observed, with overlapping regions in the outer layer. Pro/ADH@ZIF‐8‐3 and ADH/Pro@ZIF‐8‐3 show distinct core‐shell structures, where Pro or ADH is clearly positioned in separate layers through fluorescence microscopy images (Figure S24, Supporting Information).

To evaluate the cascade activity of Pro and ADH in MOF, L‐norvaline ethyl ester hydrochloride was used as the substrate, and the intermediate product L‐norvaline generated by Pro catalysis was further converted into NADH in the presence of ADH and the NAD cofactor (Figure 3b). The free enzyme concentration was calculated based on the encapsulation efficiency to ensure equivalent enzyme content for all samples (Table S1, Supporting Information). The results show that the relative activity of Pro/ADH@ZIF‐8‐1 is 1.36 times higher than that of the free enzyme. In contrast, Pro/ADH@ZIF‐8‐2 and ADH/Pro@ZIF‐8‐2 exhibit 5.36 and 4.21 times higher activity, respectively, compared to the free enzyme at the same enzymes’ concentration. Consequently, the multi‐enzyme cascade activity follows the order Pro/ADH@ZIF‐8‐2 > ADH/Pro@ZIF‐8‐2 > Pro/ADH@ZIF‐8‐1 (Figure 3f; Figure S25 and Table S2, Supporting Information). For Strategy 3, Pro/ADH@ZIF‐8‐3 and ADH/Pro@ZIF‐8‐3 exhibit 3.07 and 2.37 times the activity of the free enzyme at the same enzymes’ concentration, respectively. Both strategies 2 and 3 outperform Pro/ADH@ZIF‐8‐1, demonstrating that spatially organized encapsulation of incompatible enzymes enhances their cascade activity. Biocomposites with Pro positioned in the outer layer consistently demonstrate higher activity than those with ADH in the outer layer, consistent with the GOx‐HRP model, where the first enzyme located in the outer layer have easier access to substrates, enhancing catalytic efficiency. Furthermore, Strategy 2 exhibits higher activity compared to Strategy 3. Under harsh conditions, all three MOF composites (Pro/ADH@ZIF‐8‐1, Pro/ADH@ZIF‐8‐2, and Pro/ADH@ZIF‐8‐3) retained more than 50% of their initial activity, whereas the free enzyme rapidly lost its activity when exposed to elevated temperatures or DMSO (Figure 3g). In the recyclability tests, both Pro/ADH@ZIF‐8‐1 adn Pro/ADH@ZIF‐8‐3 biocomposites exhibited strong reusability, retaining over 50% of their initial activity after five cycles (Figure S26, Supporting Information). Notably, Pro/ADH@ZIF‐8‐2 maintained more than 90% of its initial activity after ten cycles, highlighting its exceptional stability for repeated use (Figure 3h).

### 2.4 Generalization of this Strategy to the Three‐Enzyme Cascade System

**Figure 4:**
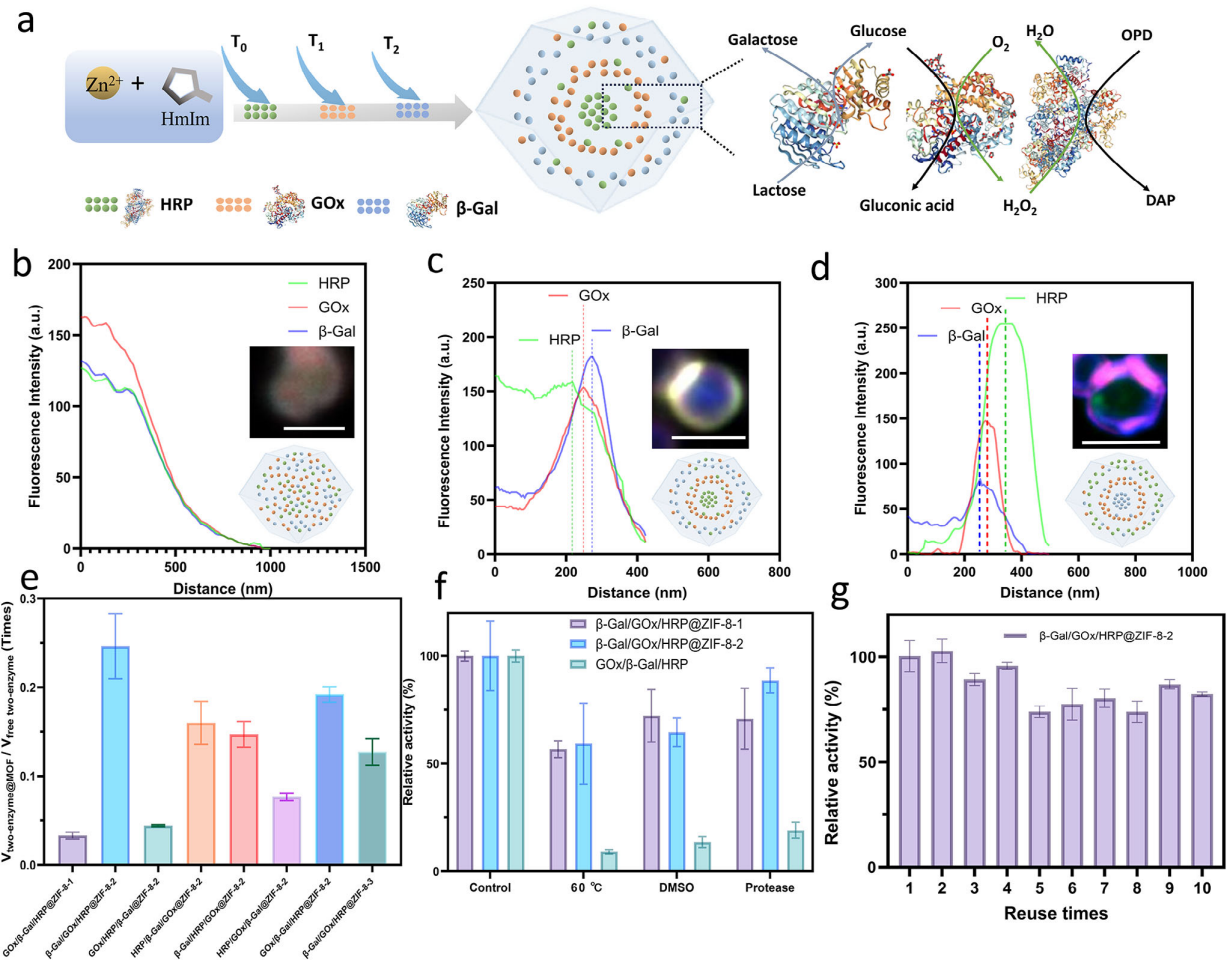
Three‐enzyme@MOF characterizations and performance. a) Schematic diagram of the three‐enzyme cascade reaction. b–d) Fluorescence intensity profile of MOF samples, calculated based on the inserted fluorescence microscopy image, their schematic images are also inserted. The scale bar is 1 µm. (b) β‐Gal/GOx/HRP@ZIF‐8‐1, (b) β‐Gal/GOx/HRP@ZIF‐8‐2, and (c) HRP/GOX/β‐Gal@ZIF‐8‐2. e) The relative activity (V_enzymes‐MOF_ and V_free enzyme_) of the initial reaction rates (V) between three‐enzyme@MOF samples and free three‐enzyme for β‐Gal/GOx/HRP model. f) The relative activity of β‐Gal/GOx/HRP@ZIF‐8‐2, β‐Gal/GOx/HRP@ZIF‐8‐1, and free β‐Gal/GOx/HRP under adverse environments. g) The relative activity of β‐Gal/GOx/HRP@ZIF‐8‐2 in ten consecutive cycles.

This method was further extended to the encapsulation of three enzymes: β‐Galactosidase (β‐Gal), GOx, and HRP, where β‐Gal first hydrolyzes lactose into glucose and galactose, GOx then oxidizes glucose to produce hydrogen peroxide, and HRP uses the hydrogen peroxide to catalyze the final reaction step. Enzymes were encapsulated in MOF via strategies 1, 2, and 3, respectively. In Strategy 1, all three enzymes were mixed and added at the start of synthesis to form a mixed biocomposite, named β‐Gal/GOx/HRP@ZIF‐8‐1. In Strategy 2, enzymes were added at 0 min, 80 min and 160 min to create a sequentially layered structure (**Figure** **4**a). β‐Gal, GOx, and HRP are layered in sequence from the outer to the inner layer, which is denoted as β‐Gal/GOx/HRP@ZIF‐8‐2. Other samples synthesized with this method were named according to their enzyme addition order: HRP/GOx/β‐Gal@ZIF‐8‐2, GOx/HRP/β‐Gal@ZIF‐8‐2, HRP/β‐Gal/GOx@ZIF‐8‐2, β‐Gal/HRP/GOx@ZIF‐8‐2, and GOx/β‐Gal/HRP@ZIF‐8‐2. In Strategy 3, HRP and MOF precursors solution were combined at the beginning, the mixture was centrifuged to remove unreacted components. Following centrifugation, a MOF precursor solution with GOx or β‐Gal was added, and synthesis continued. This layered but separated structure was named β‐Gal/GOx/HRP@ZIF‐8‐3. SEM images show the characteristic morphology of ZIF‐8 crystals (Figure S27, Supporting Information), and XRD patterns showed distinct diffraction peaks corresponding to ZIF‐8′s crystalline structure (Figure S28, Supporting Information). FTIR analysis further confirmed enzymes encapsulation, as the amide I band shifted from 1650 cm⁻¹ in the free enzyme to 1655 cm⁻¹ after immobilization within the MOFs framework (Figure S29, Supporting Information). The THz‐Far‐IR spectra further confirmed the well‐defined MOFs crystal structure, showing characteristic peaks associated with metal‐ligand interactions, which are crucial indicators of MOF integrity (Figure S30, Supporting Information).

To further verify the multi‐layer encapsulation, fluorescence microscopy was observed with fluorescein isothiocyanate (FITC)‐labelled β‐Gal, Atto 633 NHS ester labelled GOx and Atto 550 NHS ester labelled HRP. However, it was challenging to clearly observe the separation of the three colours. Therefore, fluorescence colocalization was analysed. The fluorescence image of β‐Gal/GOx/HRP@ZIF‐8‐1 showed a uniform colour distribution without any visible separation (Figure 4b; Figure S31a, Supporting Information). HRP in β‐Gal/GOx/HRP@ZIF‐8‐2 and HRP/GOX/β‐Gal@ZIF‐8‐2 are positioned in the core and shell, whereas GOx and β‐Gal appeared closer together, with a narrower distribution between their peaks (Figure 4c; Figure S31b, Supporting Information). For β‐Gal/GOx/HRP@ZIF‐8‐2, particle size rapidly increased to 768 nm at 80 min, indicating a fast coprecipitation process where the MOF framework forms quickly, while particle sizes slowly reach 893 nm at 160 min and 1039 nm at 240 min (Figures S32, Supporting Information). For Strategy 3, the enzymes‐MOF sizes reached 768, 893, and 1039 nm at 80, 160, and 240 min, respectively, indicating that the MOFs continued to grow over time rather than forming new MOFs.

The free enzyme concentration for the activity test was calculated based on the encapsulation efficiency of enzymes in MOF (Table S1, Supporting Information). The results show that the relative activity of β‐Gal/GOx/HRP@ZIF‐8‐1 is only 3.3% of the activity of its free enzyme counterparts. In contrast, β‐Gal/GOx/HRP@ZIF‐8‐2 and β‐Gal/GOx/HRP@ZIF‐8‐3 exhibit 24.6% and 12.7% of the activity of its free enzyme counterparts, respectively. Thus, the three‐enzyme cascade activity follows the order β‐Gal/GOx/HRP@ZIF‐8‐2 > β‐Gal/GOx/HRP@ZIF‐8‐3 > β‐Gal/GOx/HRP@ZIF‐8‐1. Additionally, other samples from Strategy 2 show lower activity than β‐Gal/GOx/HRP@ZIF‐8‐2, demonstrating the critical role of spatially organized encapsulation in enhancing cascade activity. (Figure 4e; Figure S33 and Table S2, Supporting Information). Samples with β‐Gal positioned in the outer layer consistently exhibit a higher activity compared to those where β‐Gal is located in the inner layer, aligning with observations in the GOx‐HRP and Pro‐ADH models. Stability experiments were conducted to evaluate the activity retention of β‐Gal/GOx/HRP@ZIF‐8‐2, β‐Gal/GOx/HRP@ZIF‐8‐1, and the free enzyme mixture (β‐Gal/GOx/HRP) under harsh conditions. When exposed to elevated temperatures (60 °C) for 1 h, DMSO for 1 h, and protease for 2 h, both enzymes‐MOF samples retained over 50% of their original activity, demonstrating significant stability compared to the free enzyme, which exhibited rapid deactivation (Figure 4f). Additionally, after five cycles, β‐Gal/GOx/HRP@ZIF‐8‐1 retained ≈60% of their initial activity (Figure S34, Supporting Information), whereas β‐Gal/GOx/HRP@ZIF‐8‐2 retained over 80% of its initial activity after ten cycles (Figure 4g).

Strategy 2 demonstrates versatility as a general approach for various multi‐enzyme cascade reactions. It is applicable not only to two‐enzyme cascades, including both compatible and incompatible enzyme systems, but also to three‐enzyme cascades. Furthermore, this strategy holds great potential for practical industrial applications, such as the synthesis of polysaccharides (e.g., maltoheptaose,^[^ ^33^ ^]^ D‐allulose^[^ ^34^ ^]^) and pharmaceutical intermediates (e.g., molnupiravir,^[^ ^35^ ^]^ islatravir,^[^ ^36^ ^]^ and artemisinin^[^ ^37^ ^]^). By providing a stable, environmentally friendly, and scalable platform, this approach facilitates efficient biocatalysis for industrial processes.

## 3 Conclusion

In conclusion, we developed a sequential strategy for spatially controlled multi‐enzyme encapsulation within MOFs without the need for intermediate isolation. Synchrotron THz‐Far‐IR analysis revealed that this process begins with coprecipitation to form a stable MOF core, followed by biomineralization to create a shell, enabling spatially positioning of enzymes in a core‐shell structure. This method, successfully applied to compatible (GOx‐HRP), incompatible (Pro‐ADH), and three‐enzyme (β‐Gal/GOx/HRP) cascades, significantly enhances catalytic efficiency, stability, and compatibility by optimizing spatial arrangements, minimizing cross‐reactivity, and facilitating intermediate transfer. The encapsulated systems demonstrate exceptional stability against temperature, proteolysis, and organic solvents, alongside excellent reusability, highlighting their strong potential for practical biocatalytic applications.

## Conflict of Interest

The authors declare no conflict of interest.

## Supporting information



Supporting Information

## Data Availability

The data that support the findings of this study are available on request from the corresponding author. The data are not publicly available due to privacy or ethical restrictions.
