# Supplementary material for: Enhancing Multi‐Enzyme Cascade Activity in Metal‐Organic Frameworks via Controlled Enzyme Encapsulation
Source: Small. 2025 Apr 8;21(22):2503059. doi: 10.1002/smll.202503059 (PMC12138836; doi:10.1002/smll.202503059)
Supplement: Supplementary file 1 — Supporting Information [file SMLL-21-2503059-s001.docx]

Supporting Information

**Enhancing Multi-Enzyme Cascade Activity in Metal-Organic Frameworks via Controlled Enzyme Encapsulation**

*Wenqing Fan^a^, Zefang Yu^a^, Dominique Appadoo^c^, Kang Liang^a,b^*, Jieying Liang,^a^**

^a^School of Chemical Engineering, Australian Centre for NanoMedicine, The University of New South Wales, Sydney, NSW 2052, Australia

^b^Graduate School of Biomedical Engineering, The University of New South Wales, Sydney, NSW 2052, Australia

^c^Australian Synchrotron-ANSTO, Clayton, Victoria, 3168 Australia

*Email: [kang.liang@unsw.edu.au](mailto:kang.liang@unsw.edu.au); [jieying.liang@unsw.edu.au](mailto:jieying.liang@unsw.edu.au)

**Experimental Section/Methods**

*Materials*: Horseradish peroxidase (HRP), glucose oxidase (GOx), β-Galactosidase (β-Gal), alkaline protease (Pro), Alcohol Dehydrogenase from Saccharomyces cerevisiae (ADH), zinc nitrate hexahydrate (99.0%), 2-methylimidazole (HmIm, 99.0%), glucose, lactose, o-phenylenediamine dihydrochloride (OPD), L-Norvaline Ethyl Ester Hydrochloride (98.0%), cofactor NAD^+^, phosphate-buffered saline (PBS), fluorescein isothiocyanate (FITC), Atto 633 NHS ester (Atto 633) and Atto 550 NHS ester (Atto 550) were purchased from Sigma-Aldrich (Australia). All other reagents were purchased from Sigma-Aldrich (Australia) and used without further purification.

*Characterizations:* Scanning electron microscopy (SEM) was carried out on an FEI Nova Nano SEM 230 with a secondary electron detector at a 10 kV acceleration voltage. The samples were pre-coated with an Emitech K575x evaporative coater before imaging. The crystallinity of biocatalytic ZIF-8 was examined using a PANalytical X-ray diffractometer (XRD). Fourier transform infrared (FTIR) spectroscopy was conducted on a Bruker Alpha FTIR Spectrometer. For fluorescence analysis, enzymes were tagged with fluorescent probes prior to encapsulation within ZIF-8 particles. Enzyme loading in the ZIF-8 samples was quantified using a fluorescent protein labeling method with the CLARIO star microplate reader (BMG LABTECH, Germany). Fluorescent images of the samples were obtained using a Zeiss LSM 900 microscope. Synchrotron terahertz and far-infrared vibrational spectroscopy (THz-Far-IR) were performed at The Australian Synchrotron (ANSTO). The metal ions content was determined by ICP-OES (Optima7000 and Avio from PerkinElmer, USA).

*Synthesis of biocatalytic ZIF-8:* Different enzyme solutions (HRP, GOx, Pro, ADH, β-Gal) (10 mg/mL) were used. The synthesis process began by mixing 40 µL of enzyme solution containing either HRP, GOx, Pro, ADH, or β-Gal at a concentration of 10 mg/mL with 45.38 mM Zn(NO_3_)·6H_2_O. Then, 861.75mM HmIm was added, and the mixture was stirred continuously. For Strategy 1, multiple enzymes were pre-mixed at the beginning of the synthesis process and reacted for 4 hours. For Strategy 2, in the two-enzyme model, the second enzyme was introduced at the 2-hour during the 4-hour stirring process. In the three-enzyme model, the second and third enzymes were added at 80 minutes and 160 minutes, respectively. For Strategy 3, the enzymes addition timing was the same as in Strategy 2. However, the mixture was centrifuged three times (6000 rpm, 5 min) at the same time points as the sample to wash off the previous MOF precursors and enzymes before adding the second or third enzyme, along with a 4.5 mM Zn(NO_3_)·6H_2_O solution and 86.2 mM HmIm. After synthesis. The final products were centrifuged at 6000 rpm for 5 minutes and washed two times with DI water to remove any remaining residues. The final nanoparticles were resuspended in 200 µL of DI water for further analysis.

*Preparation of dye-labeled enzymes:* To prepare dye-labelled enzymes, HRP and Pro were tagged with Atto 550 NHS ester, while GOx and ADH were labelled with Atto 633 NHS ester, and β-Gal was labelled with FITC. After labelling, the mixtures were allowed to stand for 5 minutes to ensure thorough interaction between the dyes and the enzymes. Excess dyes were then removed using Zeba™ Dye and Biotin Removal Spin Columns and Filter Plates, which cleared unreacted dyes and other residues via centrifugation at 1000 rcf for 2 minutes. The enzyme concentration was finally quantified to 10 mg/mL.

*Loading efficiencies of enzymes in ZIF-8:* To accurately determine the enzyme loading efficiency in ZIF-8, fluorescently labelled enzymes were used during synthesis. After the ZIF-8 formation, the encapsulated enzymes were separated by centrifugation and washed three times with water to remove any residues. The enzyme-ZIF-8 was then dissolved in the HCl solution (1M) to release the enzymes, which were quantified using their fluorescent spectra measured by a spectrophotometer. A standard curve with the same labelled enzymes was used to calculate the enzyme concentration within the MOF, and the loading efficiency was determined using equation (1).

Loading efficiency (%) =$\frac{C_{1}\times V_{1}}{m}$×100% (1)

Where C_1_ is the actual concentration of enzyme determined in the MOF after dissolution. V_1_ is the total volume after dilution in which the enzyme is quantified. m (mg) is the mass of enzyme initially added to the synthesis mixture.

*GOx and HRP cascade reaction:* To assess the enzymatic activity of GOx and HRP, a spectrophotometric assay was conducted using a microplate reader, employing o-phenylenediamine (OPD) and glucose as substrates. GOx catalyzes the oxidation of glucose by oxygen, yielding gluconic acid and hydrogen peroxide (H₂O₂) (Equation 2). The generated H₂O₂ serves as the substrate for the subsequent reaction catalyzed by HRP, which converts H₂O₂ and OPD into 2,3-diaminophenazine (DAP) (Equation 3). The reaction setup involved adding 120 µL of OPD solution (1 mg/mL) and 100 µL of glucose solution (100 mg/mL) into each well of a 96-well plate, followed by the introduction of 10 µL of enzyme-ZIF-8 sample. The mixture was thoroughly mixed, and absorbance was monitored at 420 nm. The initial linear slope, representing enzymatic activity, was calculated based on the slope of the absorbance-time curve.

Glucose + O_2_ $\underset{\to}{GOx}$ Gluconic Acid + H_2_O_2_ (2)

H_2_O_2_ + OPD $\underset{\to}{HRP}$ DAP+ H_2_O (3)

*Pro and ADH cascade reaction:* The enzymatic activity of Pro and ADH encapsulated within ZIF-8 was evaluated using a spectrophotometric assay. In this cascade, Pro catalyzes the hydrolysis of L-Norvaline Ethyl Ester, generating L-Norvaline and Ethyl Chloride, which serves as an intermediate (Equation 4). The L-Norvaline is then oxidized by ADH in the presence of NAD^+^, producing NADH, which absorbs at 340 nm (Equation 5). Each reaction mixture was prepared by adding 40 µL of PBS buffer (50 mM, pH 7.4), 110 µL of L-Norvaline Ethyl Ester Hydrochloride solution (50 mM) as the substrate for Pro, and 30 µL of NAD^+^ solution (15 mM) as a cofactor for ADH. Subsequently, 10 µL of enzyme-ZIF-8 sample was introduced, and the mixture was incubated at room temperature. The formation of NADH was monitored by measuring absorbance at 340 nm over 15 minutes using a microplate reader. The rate of NADH production provided an indication of the cascade efficiency, reflecting the activity of both enzymes.

L-Norvaline Ethyl Ester Hydrochloride$\underset{\to}{Pro}$ L-Norvaline + Ethyl Chloride (4)

L-Norvaline + NAD^+^ $\underset{\to}{ADH}$ Acetaldehyde + NADH (5)

*GOx, HRP, and β-Gal cascade reaction:* To evaluate the combined activity of β-Gal, GOx, and HRP within the MOF, a spectrophotometric assay was conducted using lactose, o-phenylenediamine (OPD), and the enzyme-ZIF-8 sample. In each well of a 96-well plate, 150 µL of lactose solution (100 mg/mL) and 50 µL of OPD solution (1 mg/mL) were added as substrates, followed by the addition of 10 µL of enzyme-ZIF-8. Absorbance was measured at 420 nm using a microplate reader to monitor the enzymatic activity. The reaction sequence begins with β-Gal catalyzing the hydrolysis of lactose into glucose and galactose, as shown in equation (6):

Lactose + H_2_O $\underset{\to}{\beta-Gal}$ Glucose + Galactose (6)

Subsequent reactions involving GOx and HRP are as same as described in section 1.6.

*Calculation of biocatalytic MOF activity:* The absorption intensity at different wavelengths reflects the bioactivity of the enzyme-ZIF-8 biocomposites. To accurately evaluate the bioactivity of free enzymes, the amount of free enzymes introduced was adjusted to match the enzyme content encapsulated in enzyme-ZIF-8 (Table S1). Enzyme activity (V_enzymes-MOF_ and V_free enzyme_) was determined by calculating the initial linear slope of the absorbance vs. time curve at a specific wavelength. The relative activity (V_enzymes-MOF_/V_free enzyme_​) was obtained by dividing the activity of enzyme-MOF by that of the free enzyme.

*Evaluation of operational stability of enzymes-MOF biocomposites:* To assess the operational stability of enzymes-MOF biocomposites, samples were subjected to various adverse conditions. Each sample was incubated at 60°C for one hour to test thermal stability, exposed to DMSO for an hour for chemical stability, and treated with Pro for two hours. A control group was maintained under normal conditions. Enzyme activity was measured after each treatment using consistent substrate and assay conditions for comparison.

For recyclability testing, enzymes-MOFs were used repeatedly in a standard reaction setup. After each reaction cycle, the mixture was filtered, and the reaction products were collected. The MOFs were washed with DI water and reused in the next cycle. This process was repeated multiple times, with enzymatic activity measured after each cycle.

**Tables and Figures**

**Table S1.** Enzyme loading efficiency of biocomposites.

| **GOx/HRP model** | | **GOx (%)** | **HRP (%)** | | |  |
| --- | --- | --- | --- | --- | --- | --- |
| GOx/HRP@ZIF-8-1 | | 6.8±0.34 | 40.40±1.72 | | |  |
| HRP/GOx@ZIF-8-2 | | 9.40±0.32 | 40.00±0.80 | | |  |
| GOx/HRP@ZIF-8-2 | | 6.00±0.09 | 36.00±0.53 | | |  |
| HRP/GOX@ZIF-8-3 | | 11.60±2.83 | 82.00±7.27 | | |  |
| GOx/HRP@ZIF-8-3 | | 15.20±0.48 | 53.00±1.57 | | |  |
| **Pro/ADH model** | | **Pro (%)** | **ADH (%)** | | |  |
| Pro/ADH@ZIF-8-1 | | 10.80±0.20 | 18.15±0.20 | | |  |
| ADH/Pro@ZIF-8-2 | | 12.45±0.14 | 3.75±0.16 | | |  |
| Pro/ADH@ZIF-8-2 | | 3.45±0.34 | 19.35±0.79 | | |  |
| ADH/Pro@ZIF-8-3 | | 7.80±0.18 | 6.15±0.21 | | |  |
| Pro/ADH@ZIF-8-3 | | 8.40±0.45 | 34.8 ±1.50 | | |  |
| **GOx/HRP/β-Gal model** | **GOx (%)** | | **HRP (%)** | **β-Gal (%)** |  |  |
| GOx/HRP/β-Gal@ZIF-8-1 | 36.25±0.15 | | 47.50±1.90 | 27.30±1.09 |  |  |
| β-Gal/GOx/HRP@ZIF-8-2 | 9.70±0.04 | | 40.95±1.64 | 11.90±0.84 |  |  |
| HRP/GOx/β-Gal@ZIF-8-2 | 2.50±0.01 | | 4.75±0.012 | 21.00±1.28 |  |  |
| GOx/HRP/β-Gal@ZIF-8-2 | 9.50±0.04 | | 14.00±0.56 | 32.03±0.28 |  |  |
| HRP/β-Gal/GOx@ZIF-8-2 | 14.50±0.06 | | 11.50±0.05 | 7.00±0.68 |  |  |
| β-Gal/HRP/GOx@ZIF-8-2 | 15.00±0.06 | | 18.25±0.73 | 4.20±0.47 |  |  |
| GOx/β-Gal/HRP@ZIF-8-2 | | 6.60±0.03 | 8.50±0.03 | 12.95±1.58 | | |
| β-Gal/GOx/HRP@ZIF-8-3 | | 7.85±0.12 | 28.92±2.16 | 12.81±0.62 | | |

**Table S2** Relative activity of enzymes-MOF versus free enzymes.

| **Biocomposites** | **V_enzymes-MOF_**  **(h^-1^)** | **V_free_ _enzyme_**  **(h^-1^)** | **Relative activity**  **(V_enzymes-MOF_/V_free_ _enzyme_)** |
| --- | --- | --- | --- |
| **GOx/HRP model** |  |  |  |
| GOx/HRP@ZIF-8-1 | 7.47±0.92 | 15.442±1.85 | 0.25±0.03 |
| HRP/GOx@ZIF-8-2 | 24.00±1.00 | 12.75±0.72 | 0.73±0.01 |
| GOx/HRP@ZIF-8-2 | 29.06±0.90 | 12.83±0.87 | 0.93±0.08 |
| HRP/GOX@ZIF-8-3 | 11.88±1.50 | 15.87±0.79 | 0.17±0.02 |
| GOx/HRP@ZIF-8-3 | 21.04±3.87 | 14.25±0.46 | 0.35±0.06 |
| **Pro/ADH model** |  |  |  |
| Pro/ADH@ZIF-8-1 | 0.17±0.02 | 0.13±0.04 | 1.36±0.03 |
| ADH/Pro@ZIF-8-2 | 0.26±0.02 | 0.06±0.01 | 4.21±0.33 |
| Pro/ADH@ZIF-8-2 | 0.40±0.05 | 0.07±0.01 | 5.36±0.78 |
| ADH/Pro@ZIF-8-3 | 0.22±0.02 | 0.09±0.06 | 2.37±0.06 |
| Pro/ADH@ZIF-8-3 | 0.24±0.01 | 0.08±0.03 | 3.07±0.34 |
| **GOx/HRP/****β-Gal model** |  |  |  |
| GOx/HRP/β-Gal@ZIF-8-1 | 0.22±0.03 | 6.82±0.05 | 0.033±0.04 |
| β-Gal/GOx/HRP@ZIF-8-2 | 0.38±0.06 | 1.56±0.11 | 0.246±0.04 |
| HRP/GOx/β-Gal@ZIF-8-2 | 0.09±0.01 | 1.17±0.15 | 0.077±0.04 |
| GOx/HRP/β-Gal@ZIF-8-2 | 0.15±0.04 | 3.49±0.20 | 0.044±0.01 |
| HRP/β-Gal/GOx@ZIF-8-2 | 0.20±0.03 | 1.22±0.02 | 0.16±0.02 |
| β-Gal/HRP/GOx@ZIF-8-2 | 0.11±0.01 | 0.75±0.03 | 0.147±0.02 |
| GOx/β-Gal/HRP@ZIF-8-2 | 0.20±0.01 | 1.05±0.05 | 0.192±0.01 |
| β-Gal/GOx/HRP@ZIF-8-3 | 0.19±0.02 | 1.52±0.19 | 0.127±0.02 |

**Table S3** Porosity characteristics of enzymes-MOF composites determined from N₂ adsorption isotherms.

| **Biocomposites** | **Surface Area**  **(m²/g)** | **Average Pore Diameter**  **(nm)** |
| --- | --- | --- |
| GOx/HRP@ZIF-8-1 | 1374.66 | 1.36 |
| GOx/HRP@ZIF-8-2 | 1919.55 | 1.28 |

**
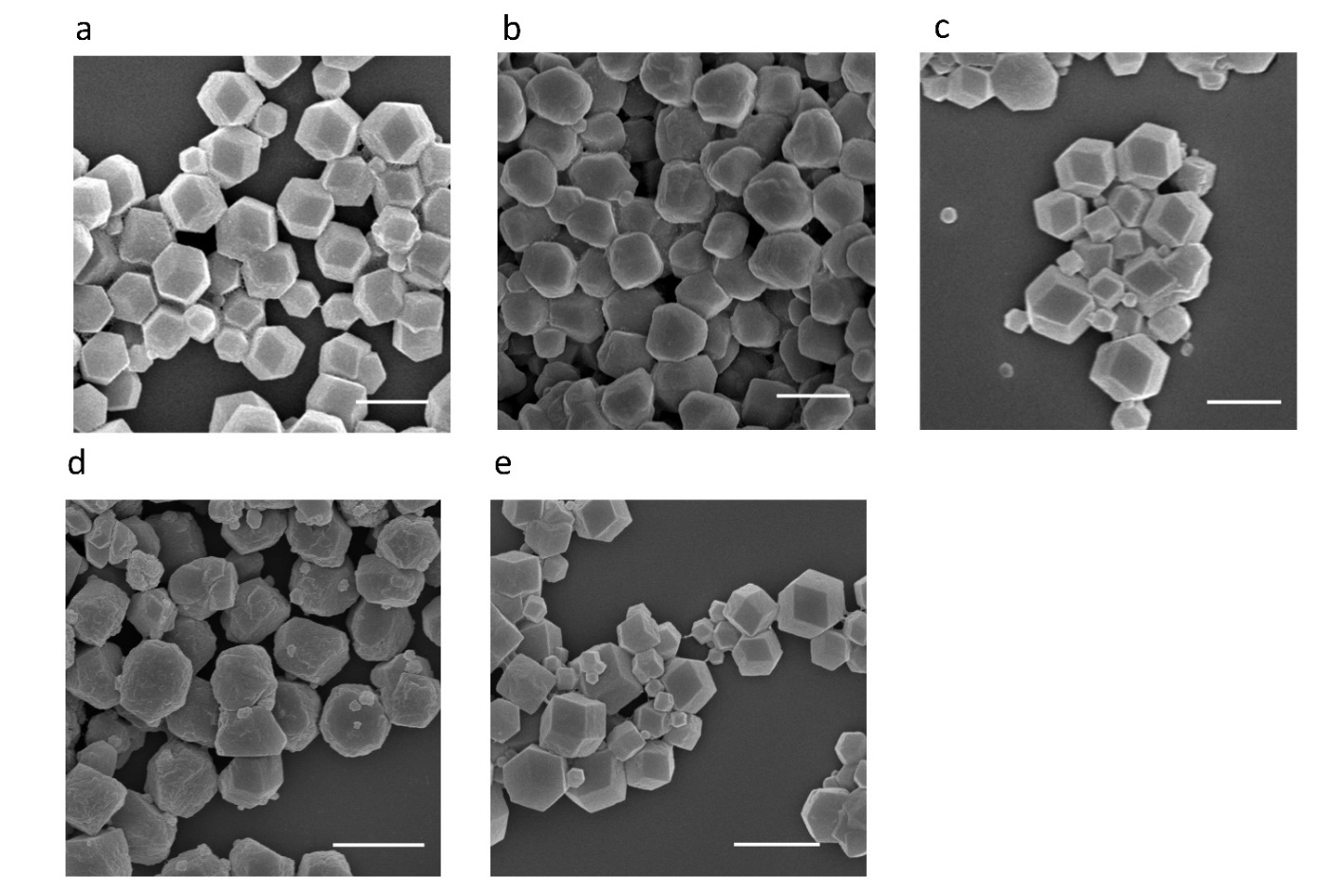
Figure S1.** SEM images of **(a)** GOx/HRP@ZIF-8-1, **(b)** GOx/HRP@ZIF-8-2, **(c)** HRP/GOx@ZIF-8-2, **(d)** GOx/HRP@ZIF-8-3, **(e)** HRP/GOx@ZIF-8-3. The scale bar is 1 µm.


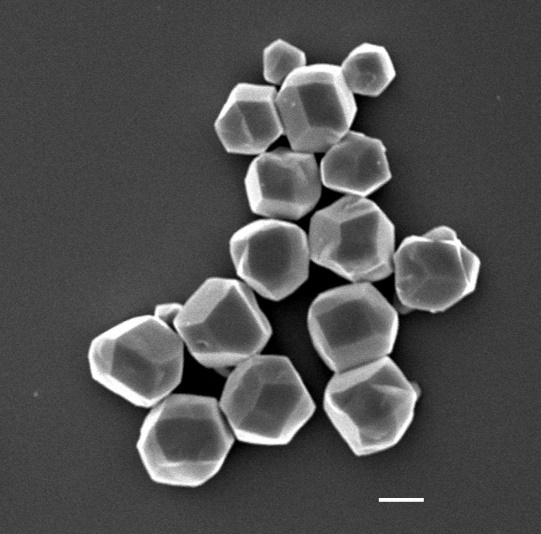


**Figure S2.** SEM images of ZIF-8. The scale bar is 1 µm.


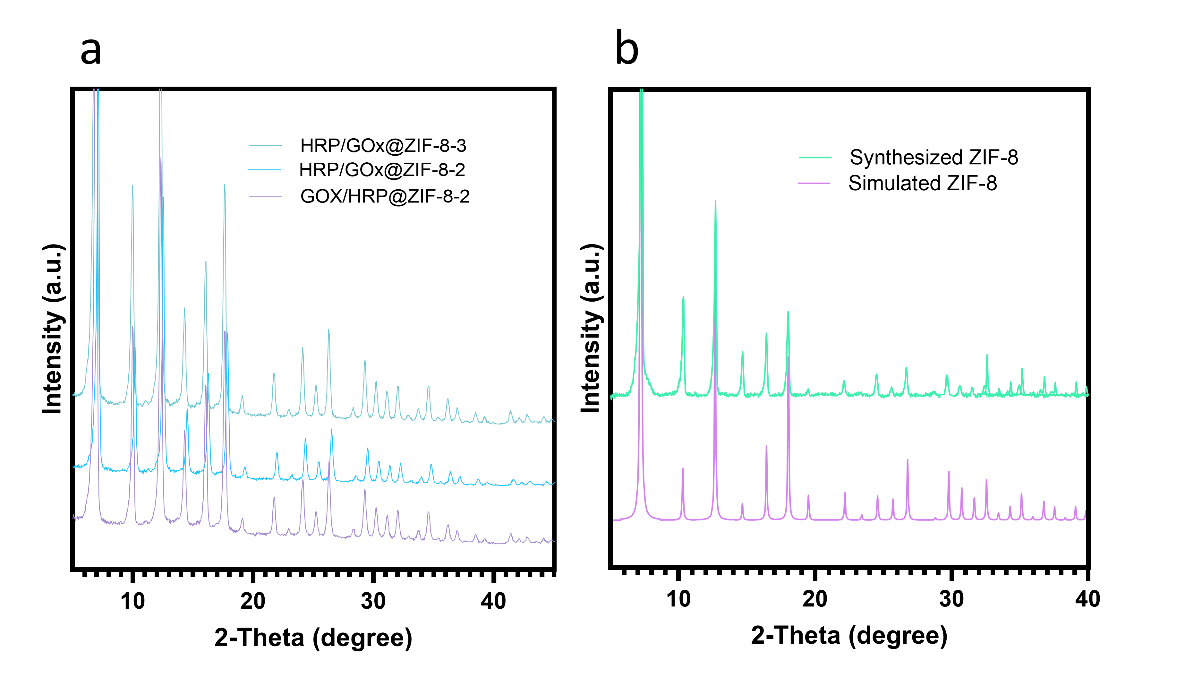


**Figure S3.** XRD patterns of (a) HRP/GOx@ZIF-8-2, GOx/HRP@ZIF-8-2, and HRP/GOx@ZIF-8-3; (b) synthesized ZIF-8 and simulated ZIF-8.

**Figure S4.** The FTIR spectra of GOx-HRP samples with dashed lines marking the amide I and II bands.


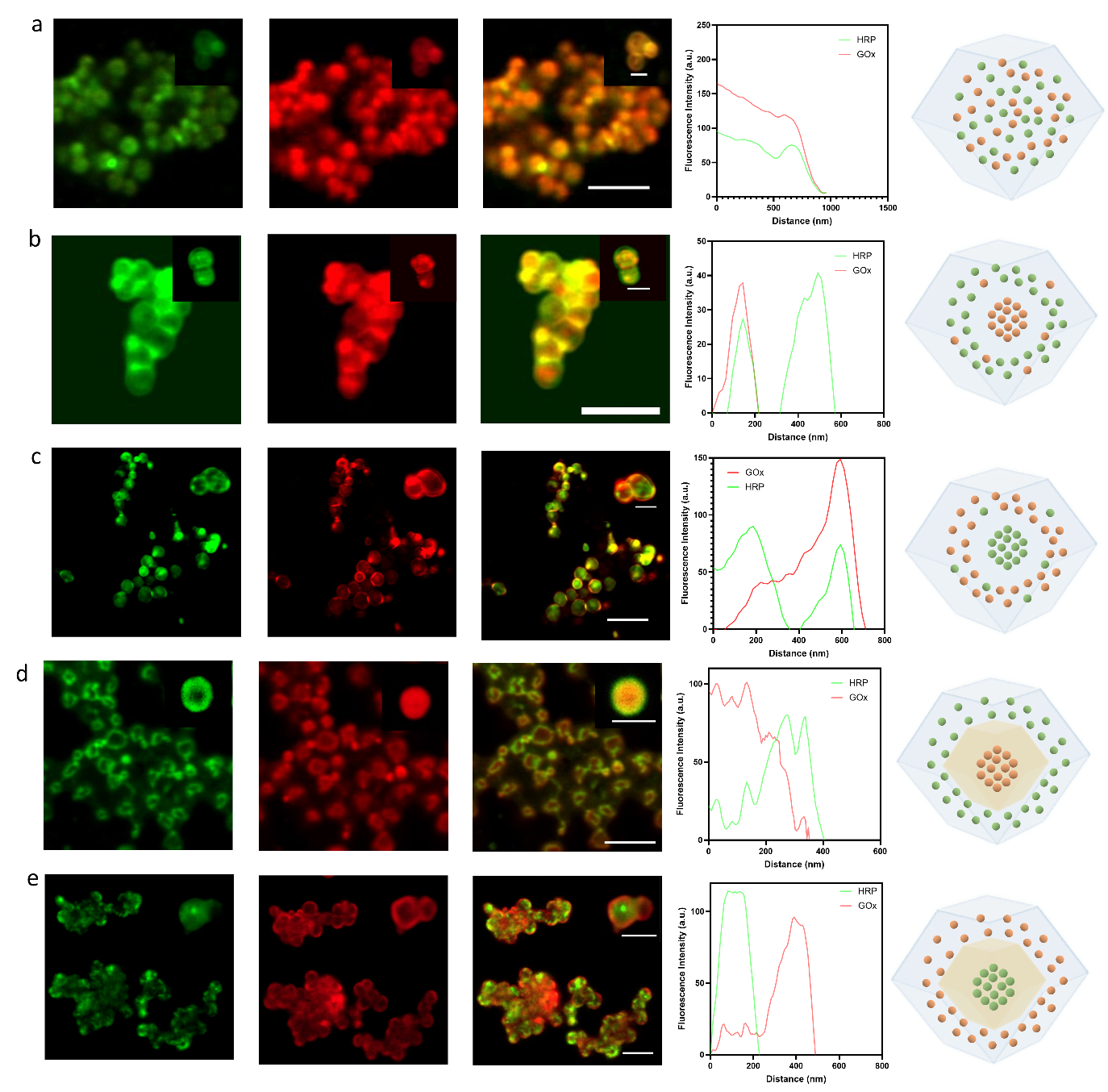


**Figure S5.** The CLSM images display the following samples: (a) GOx/HRP@ZIF-8-1, (b) HRP/GOx@ZIF-8-2, (c) GOx/HRP@ZIF-8-2, (d) HRP/GOx@ZIF-8-3, (e) GOx/HRP@ZIF-8-3. The scale bar in the main images is 3 μm, while in the top-right inset images is 1 μm. The green and red channels represent HRP and GOx fluorescence, respectively. The right image in each row shows a fluorescence intensity profile analyzed by ImageJ, and their schematic images.

**Figure S6.** Loading efficiency of HRP in the GOx/HRP@ZIF-8-2 after reaction for 2 hours and 4 hours.


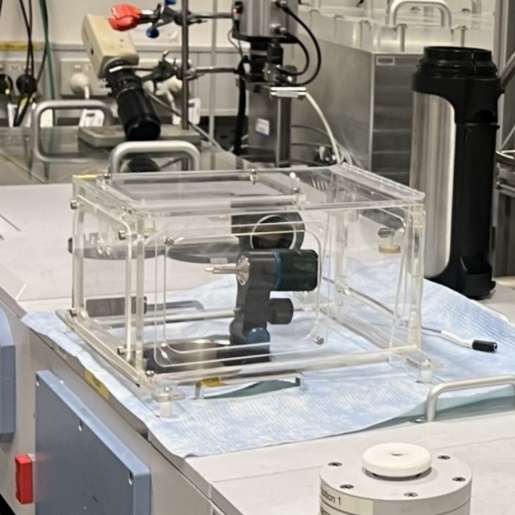

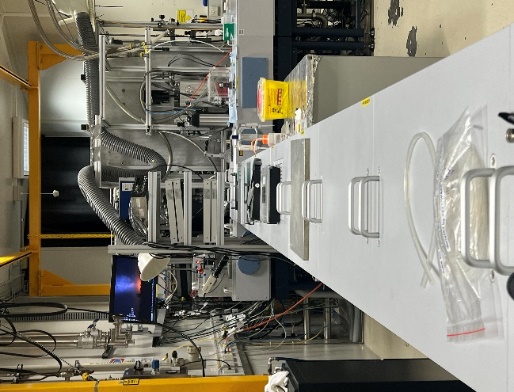

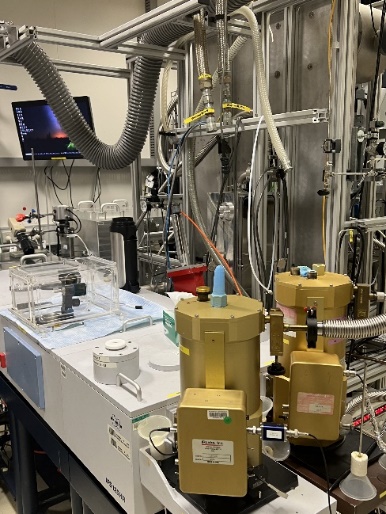


**Figure S7.** Experimental setup of THz-Far-IR experiments at Australian Synchrotron.

**Figure S8.** The THz-Far-IR spectra of ZIF-8, Zn(NO_3_)_2_·6H_2_O, and HmIm, confirming the MOF formation.

**Figure S9.** The THz-Far-IR spectra of GOx/HRP@ZIF-8-1, HRP/GOx@ZIF-8-2, GOx/HRP@ZIF-8-2, HRP/GOx@ZIF-8-3, GOx/HRP@ZIF-8-3.


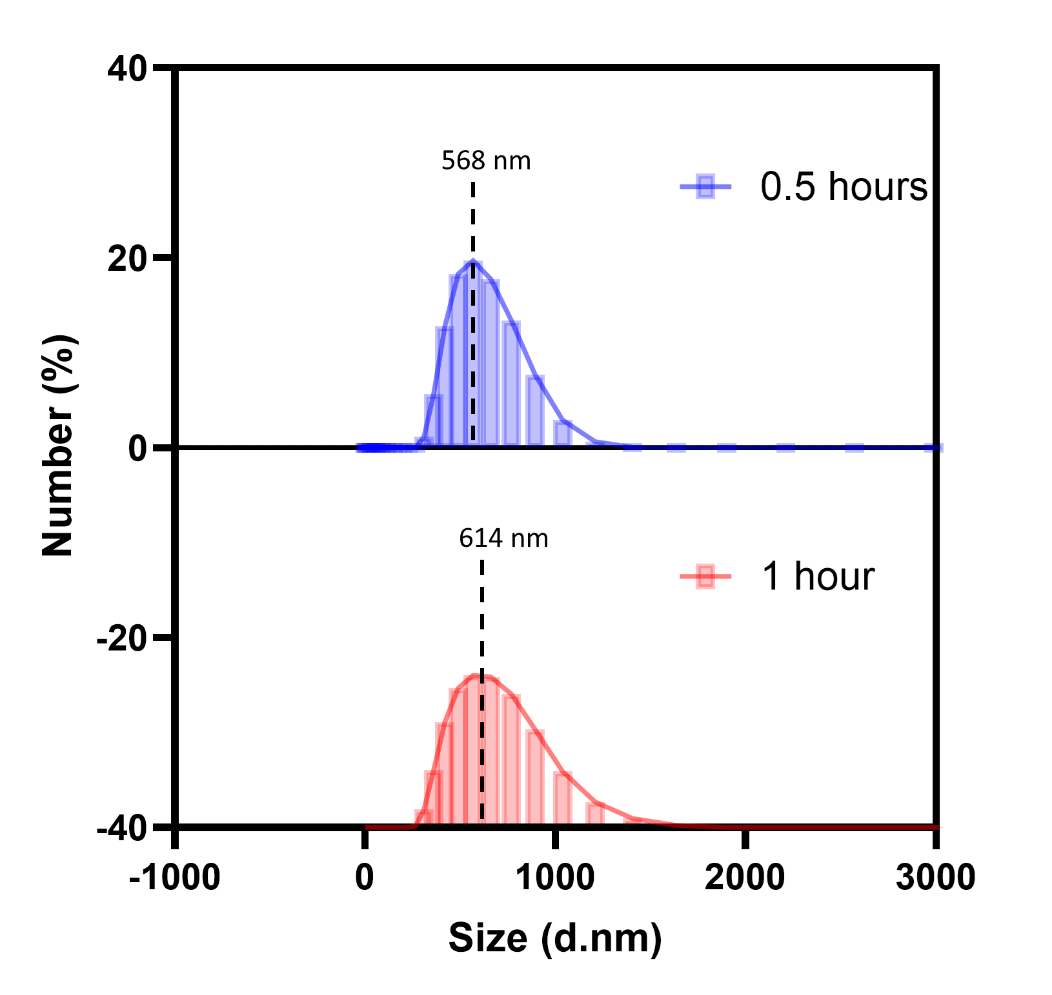


**Figure S10**. DLS analysis of GOx/HRP@ZIF-8-2 showing particle size distribution at 0.5 hour and 1 hour during synthesis.


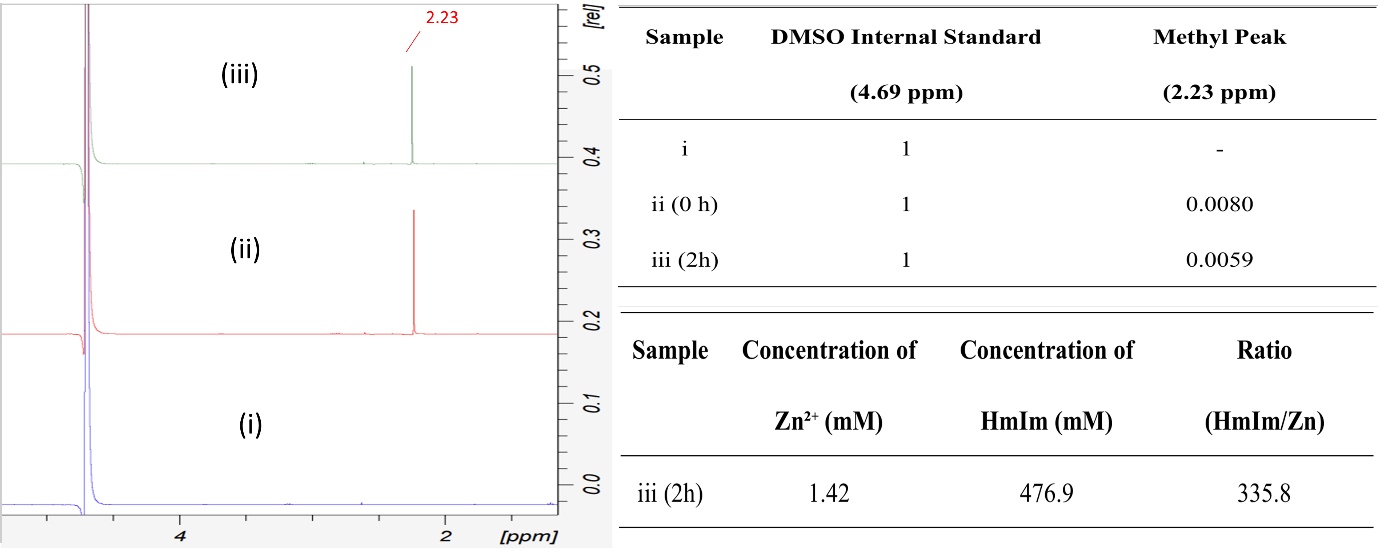


**Figure S11.** NMR spectra, (i) DMSO as the internal standard, (ii) the initial HmIm before synthesis, (iii) the mixture solution after 2-hour reaction, where the peak at 2.23 ppm represents the methyl group of HmIm. The Zn^2+^ concentration was determined by ICP.

**Figure S12.** THz-Far-IR spectra of Strategy 2, where only pre-formed MOFs were removed after prior 2-hour reaction by centrifugation at 6000 rpm for 5 min, and the second enzyme was added.

**
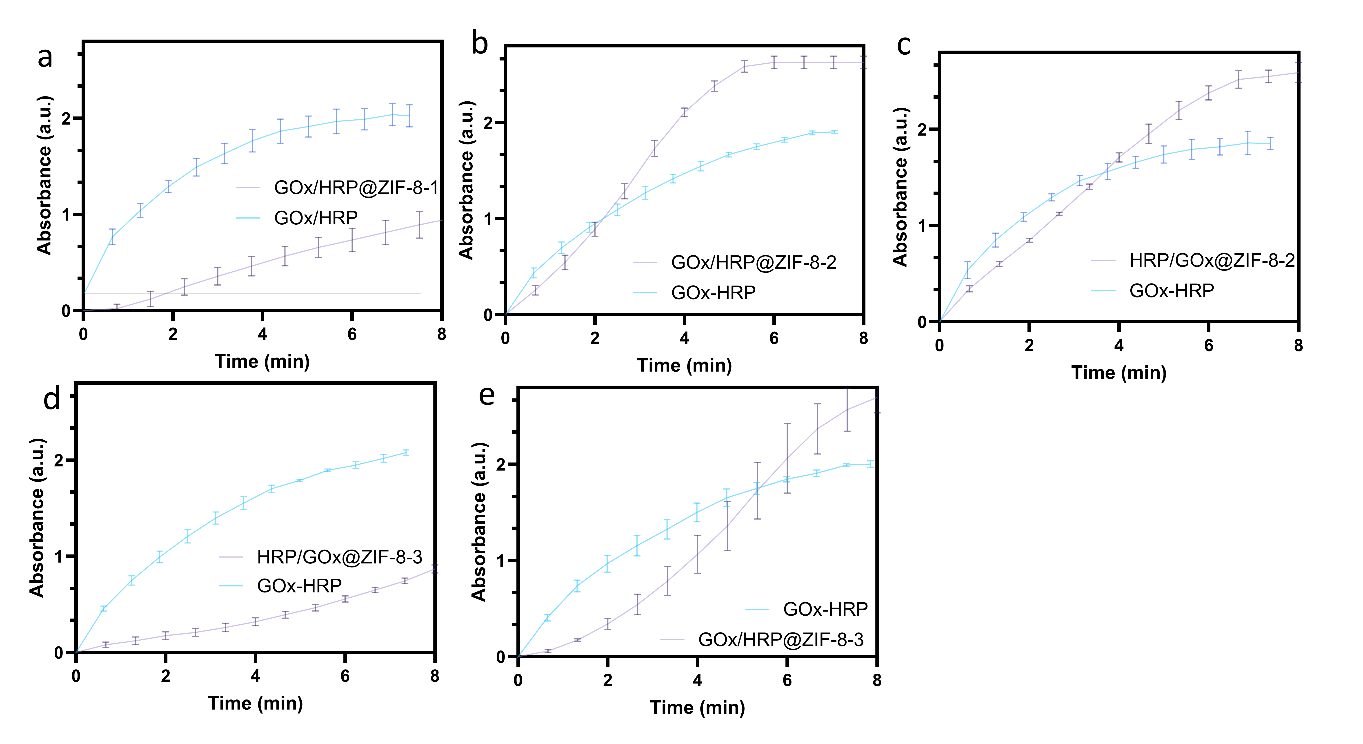
**

**Figure S13.** The comparison of enzymatic activity of two-enzyme@MOF biocomposites and their free counterparts. (a) GOx/HRP@ZIF-8-1, (b) GOx/HRP@ZIF-8-2, (c) HRP/GOx@ZIF-8-2, (d) HRP/GOx@ZIF-8-3. (e) GOx/HRP@ZIF-8-3. The same amount of free enzymes was used for comparison based on the loading efficiency. Due to the rapid reaction rate of free enzymes, the initial reaction rate is calculated within the first 2 minutes.

*
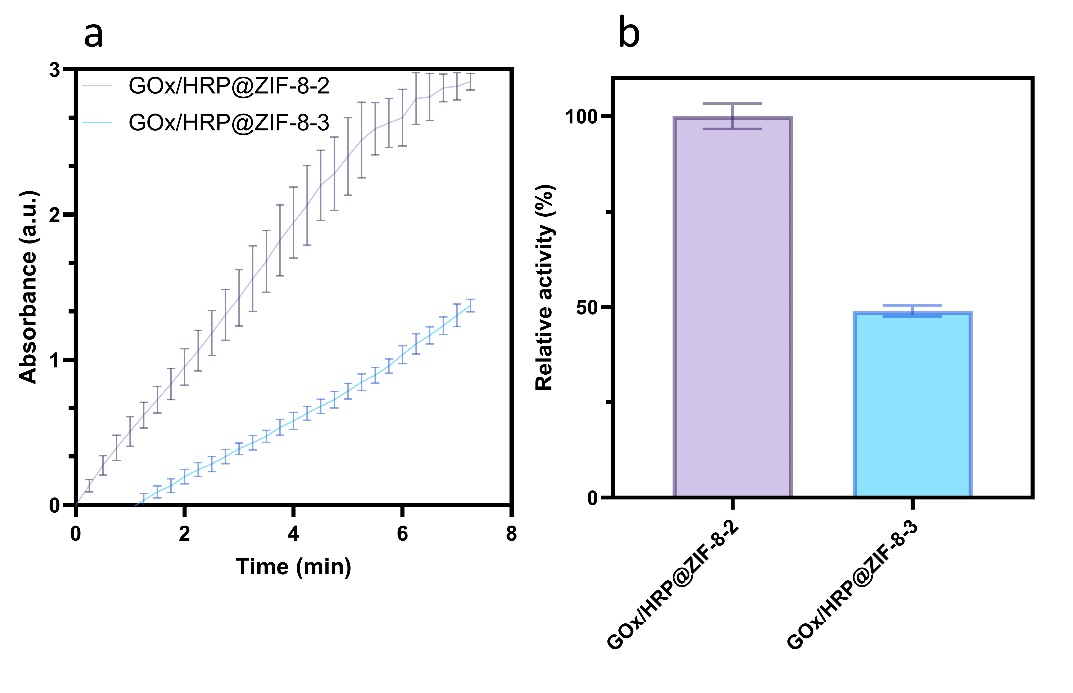
*

**Fig S14.** The trend over time (a) and relative activities (b) of GOx/HRP@ZIF-8-2 and GOx/HRP@ZIF-8-3 under identical MOF precursor concentrations.


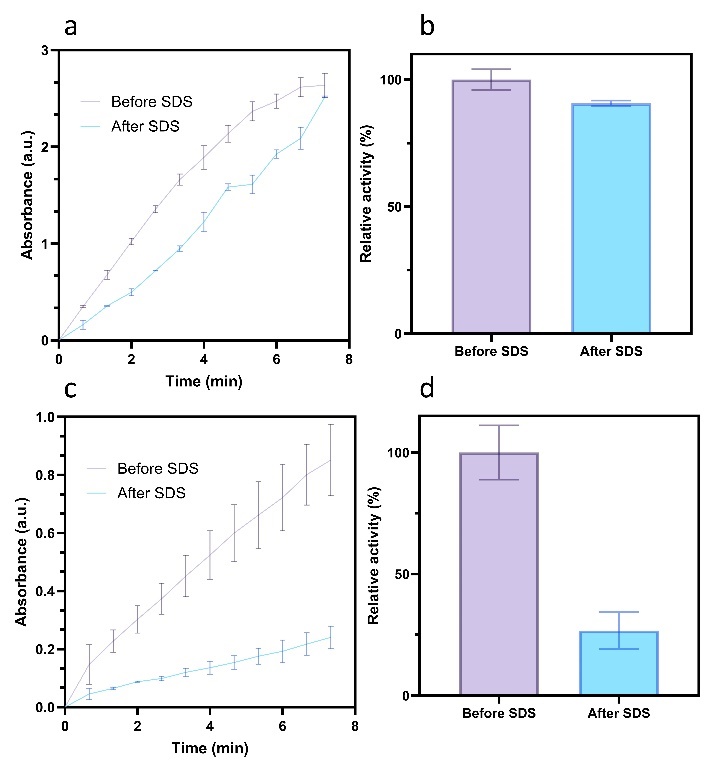


**Figure S15.** Comparison of enzyme activity before and after washing by sodium dodecyl sulphate (SDS). The trend over time of (a) GOx/HRP@ZIF-8-2 and (c) HRP-GOx-adsorbed ZIF-8. The relative activities of GOx/HRP@ZIF-8-2 (b) and HRP-GOx-adsorbed (d), with the original activity as 100%.


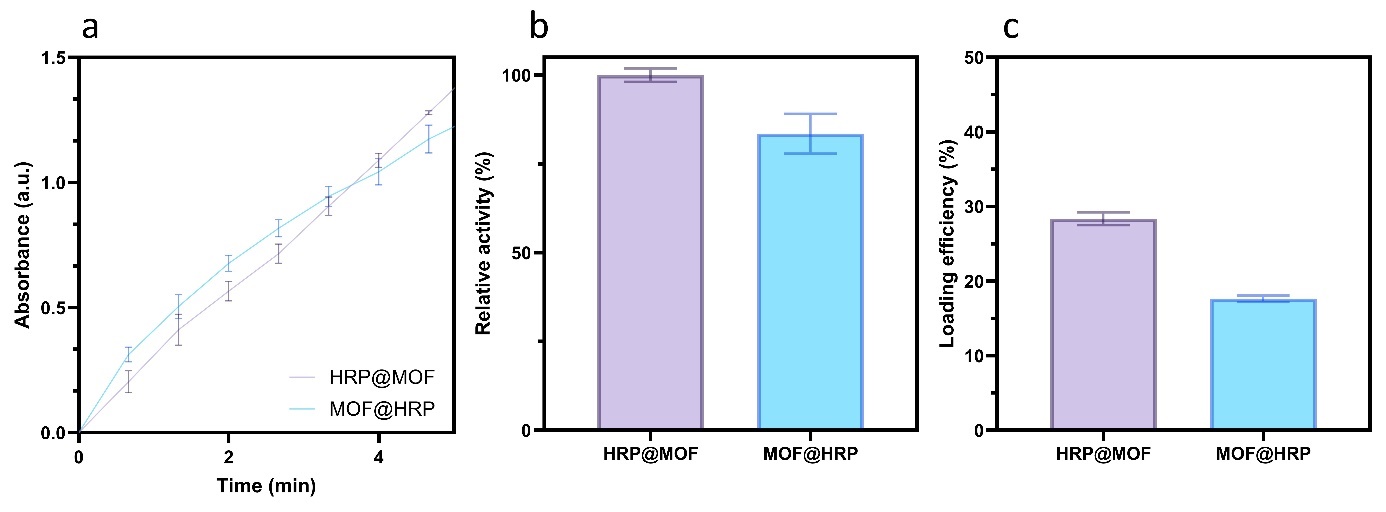


**Figure S16.** Comparison of HRP@MOF and MOF@HRP. (a) Absorbance over time based on the same enzyme amount. (b) The relative activities of HRP@MOF and MOF@HRP based on the same enzyme amount, with the activity HRP@MOF as 100%. (c) HRP loading efficiency for HRP@MOF and MOF@HRP.


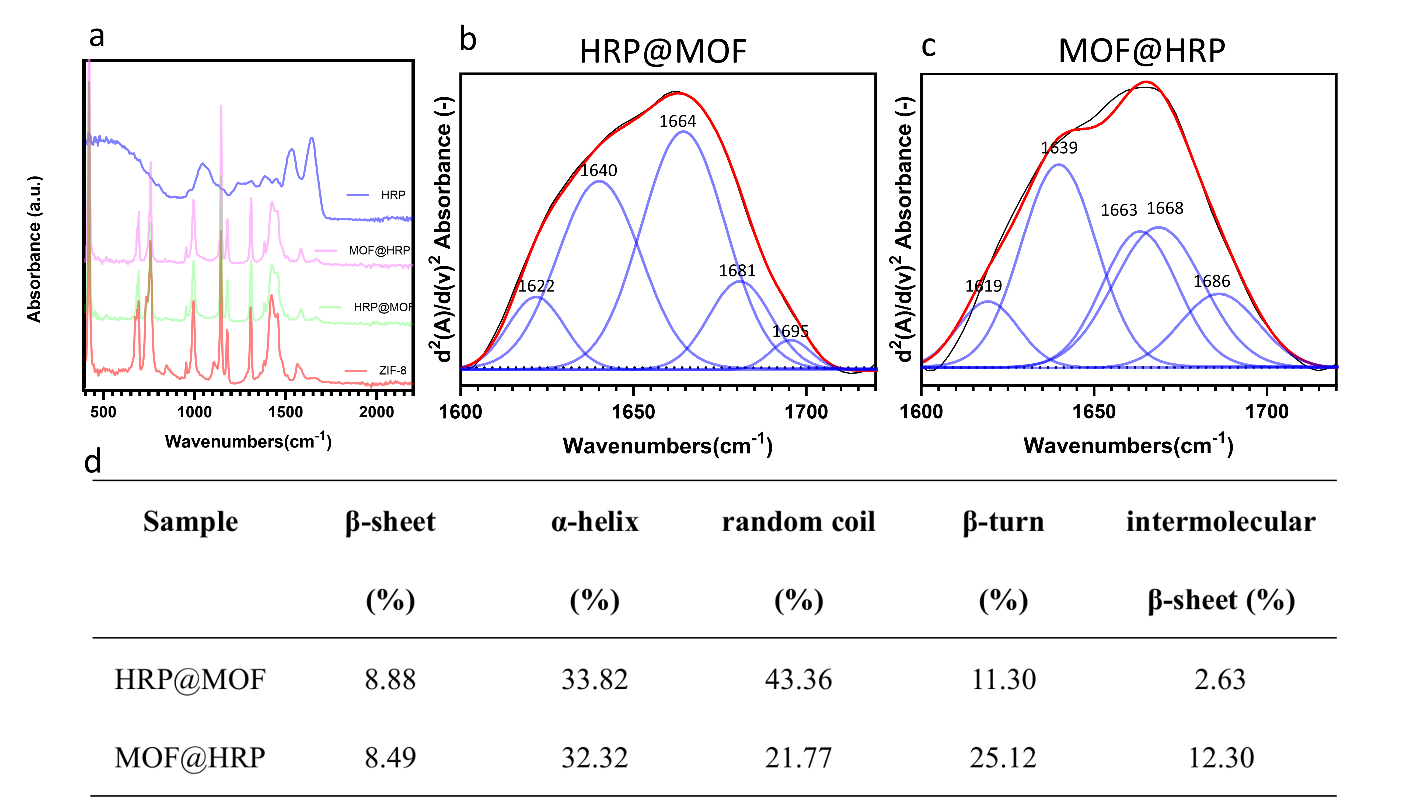


**Figure S17.** FTIR spectra and deconvolution analysis of HRP@MOF and MOF@HRP. (a) The overall FTIR spectra of free HRP, MOF@HRP, HRP@MOF, and ZIF-8. (b) and (c) show the deconvoluted amide I region for HRP@MOF and MOF@HRP. (d) The quantitative analysis of β-sheet, α-helix, intermolecular β-sheet, β-turn, and random coil structures based on the (b) and (c).

**Figure S18.** Nitrogen adsorption-desorption isotherms for GOx/HRP@ZIF-8-1 and GOx/HRP@ZIF-8-2.

**Fig S19.** The reusability test of GOx/HRP@ZIF-8-1 and GOx/HRP@ZIF-8-3 in five consecutive cycles.


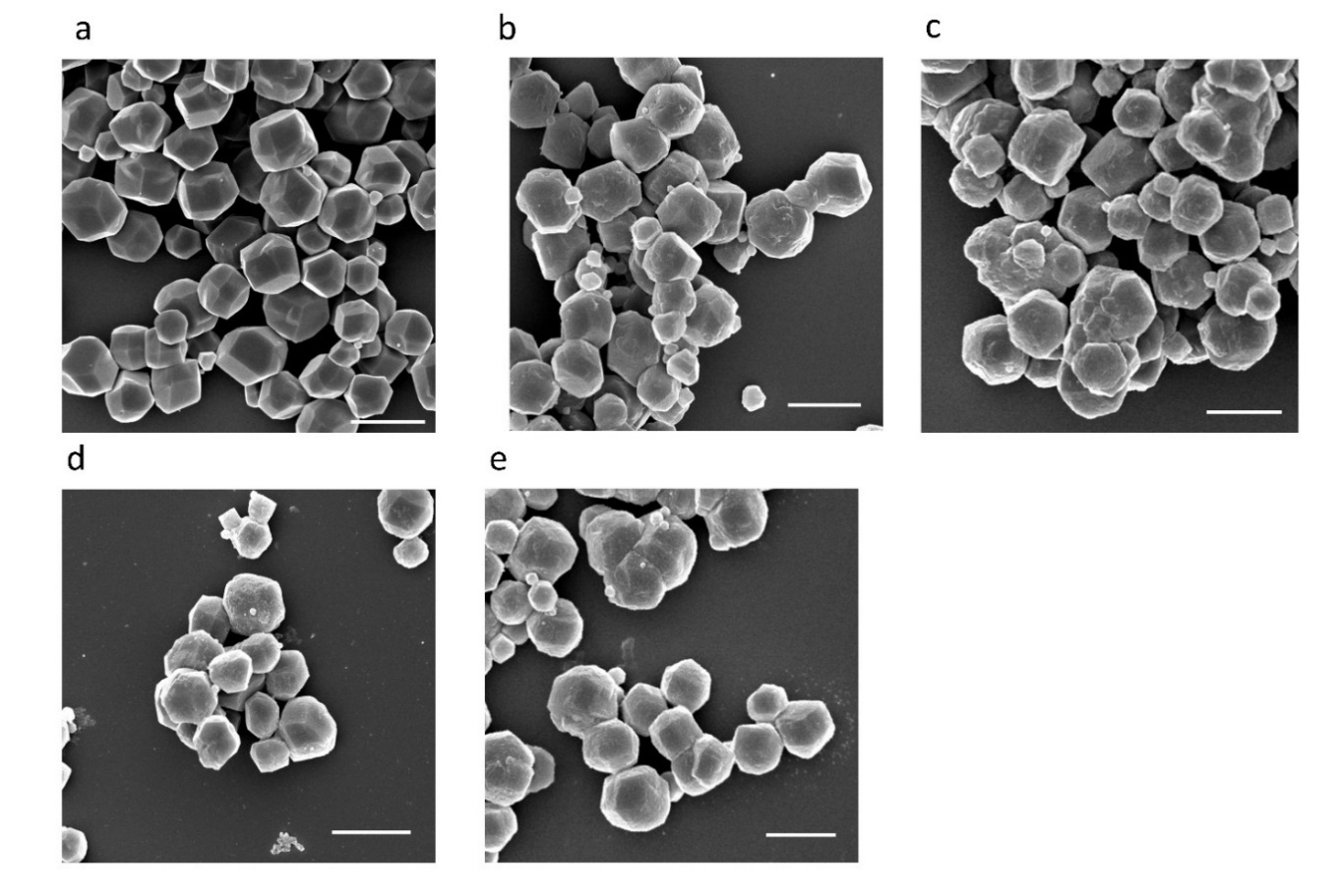


**Figure S20.** SEM images of **(a)** Pro/ADH@ZIF-8-1, **(b)** Pro/ADH@ZIF-8-2, **(c)** ADH/Pro@ZIF-8-2, **(d)** Pro/ADH@ZIF-8-3, **(e)** ADH/Pro@ZIF-8-3. The scale bar is 1 µm.

**Figure S21.** XRD patterns of Pro/ADH@ZIF-8-1, Pro/ADH@ZIF-8-2, ADH/Pro@ZIF-8-2, Pro/ADH@ZIF-8-3, and ADH/Pro@ZIF-8-3.

**Figure S22.** The THz-Far-IR spectra of Pro/ADH@ZIF-8-1, Pro/ADH@ZIF-8-2, ADH/Pro@ZIF-8-2, Pro/ADH@ZIF-8-3, ADH/Pro@ZIF-8-3.


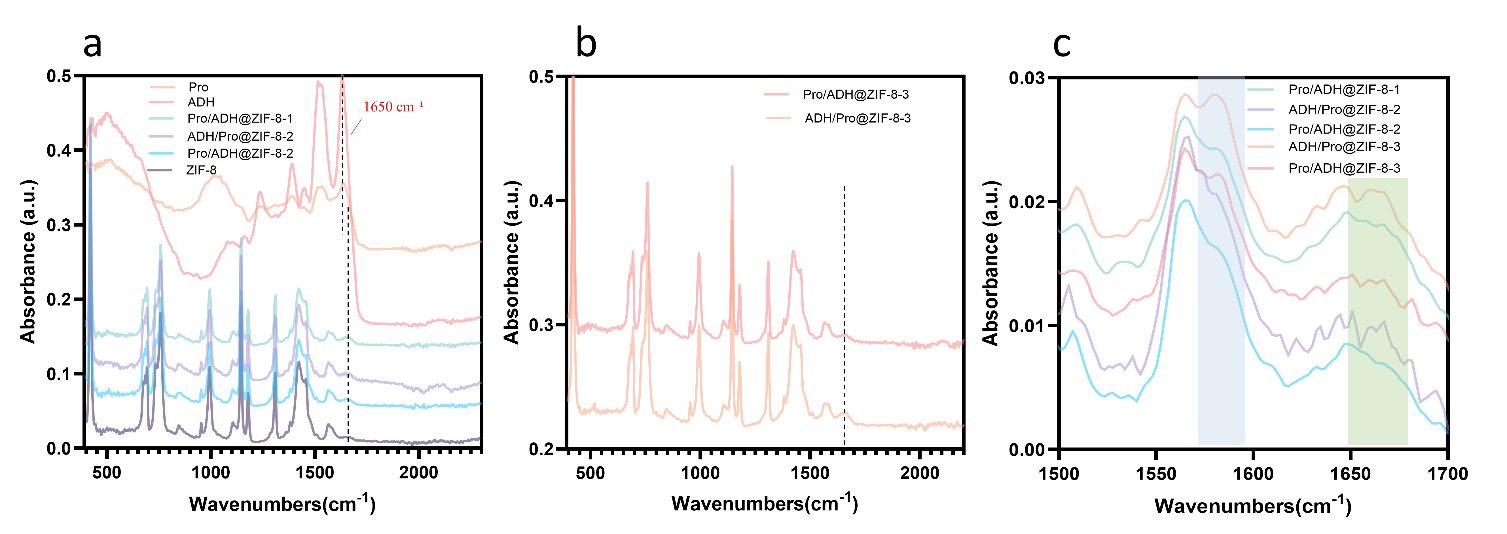


**Figure S23.** The FTIR spectra of ADH-Pro samples. (a-b) The full spectra with dashed lines marking the amide I band.

**
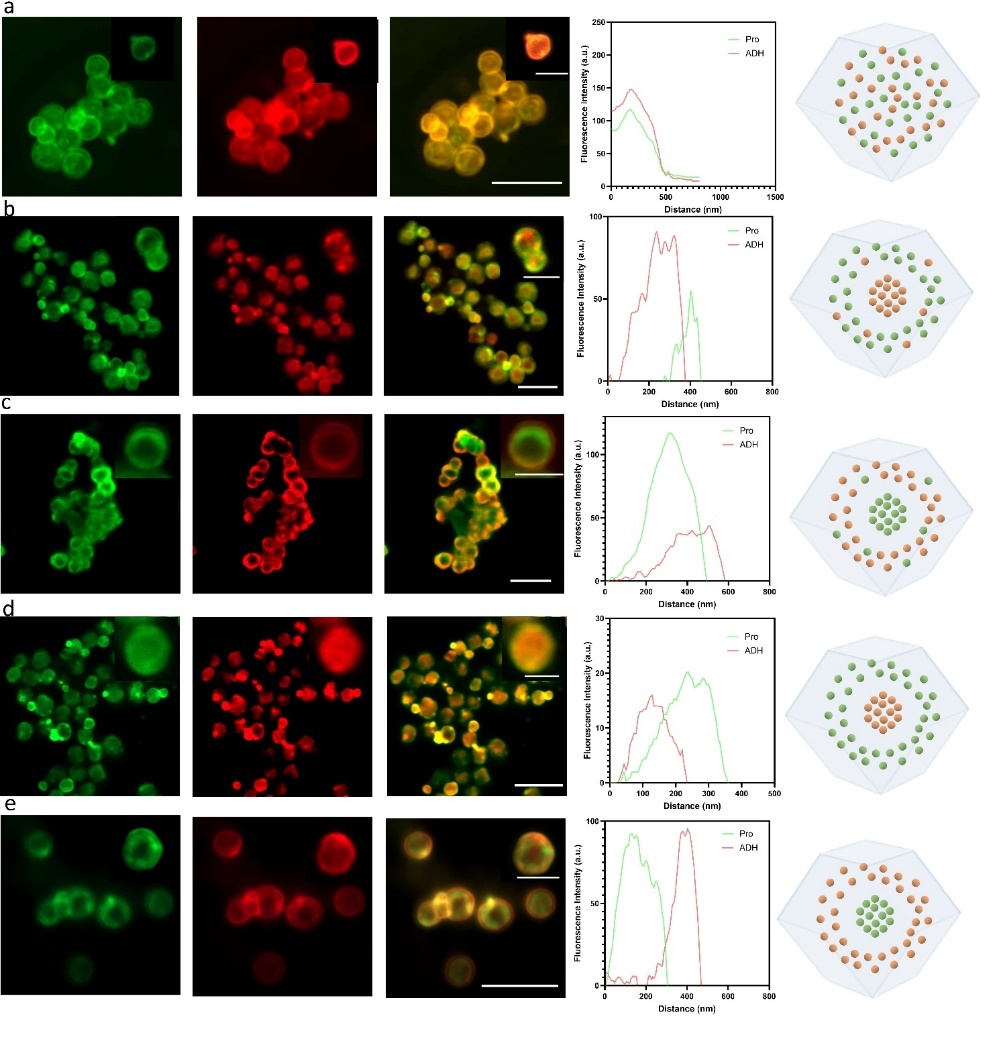
**

**Figure S24.** The CLSM images display the following samples: (a) Pro/ADH@ZIF-8-1, (b) Pro/ADH@ZIF-8-2, (c) ADH/Pro@ZIF-8-2, (d) Pro/ADH@ZIF-8-3, (e) ADH/Pro@ZIF-8-3. The scale bar in the main images is 3 μm, while in the top-right inset images, it is 1 μm. The green and red channels represent Pro and ADH fluorescence, respectively. The right right image in each row shows a fluorescence intensity profile analyzed by ImageJ, and the schematic images.


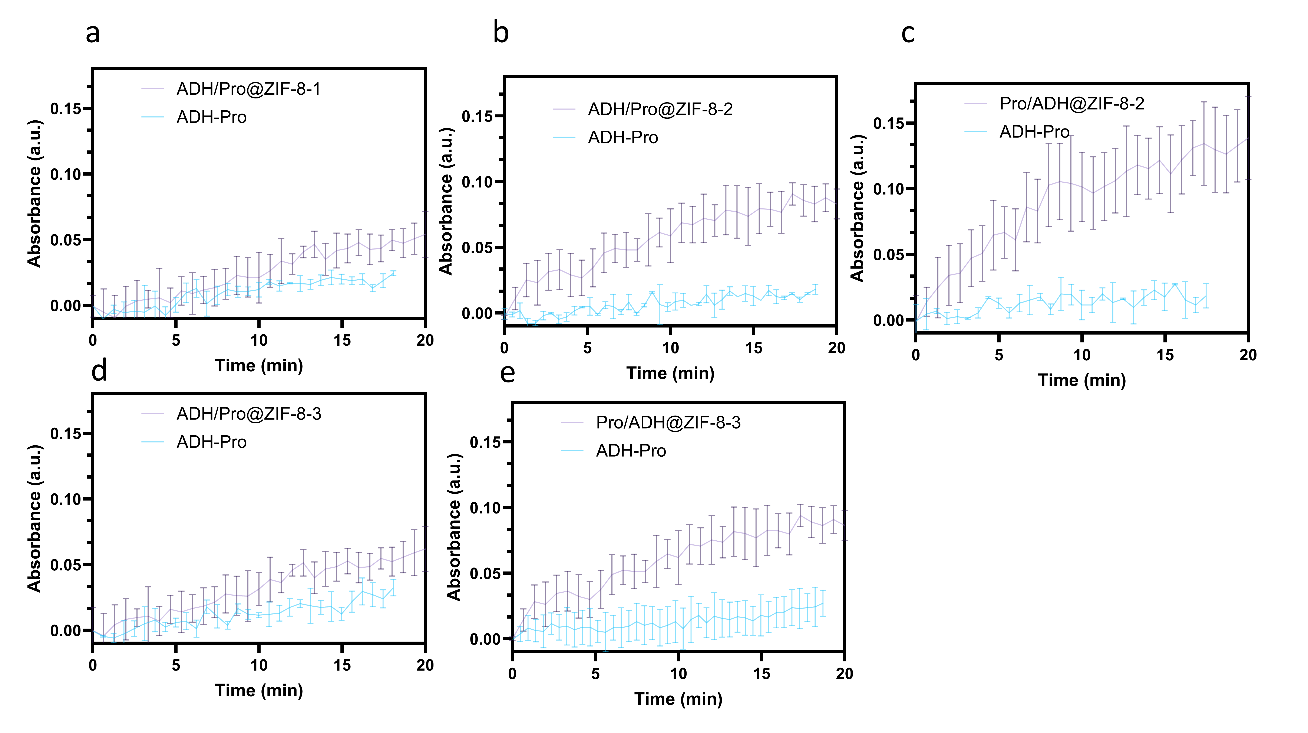


**Figure S25.** The comparison of enzymatic activity of incompatible enzymes@MOF composites and their free counterparts. (a) Pro/ADH@ZIF-8-1, (b) ADH/Pro@ZIF-8-2, (c) Pro/ADH@ZIF-8-2, (d) ADH/Pro@ZIF-8-3, (e) Pro/ADH@ZIF-8-3. The same amount of free enzymes was used for comparison based on the loading efficiency.

**Fig S26.** The reusability test of Pro/ADH@ZIF-8-1 and Pro/ADH@ZIF-8-3 in five consecutive cycles.


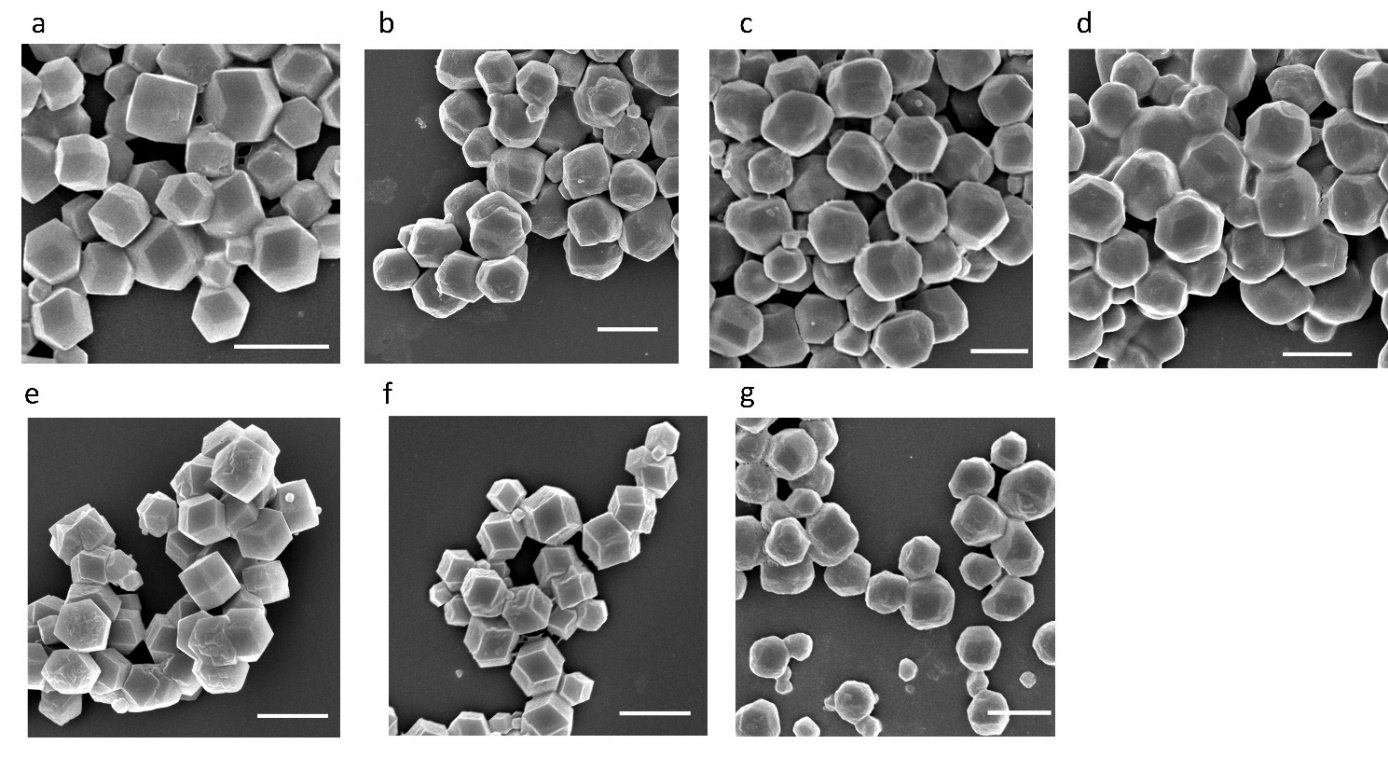


**Figure S27.** SEM images of **(a)** GOx/β-Gal/HRP@ZIF-8-1, **(b)** β-Gal/GOx/HRP@ZIF-8-2, **(c)** HRP/GOx/β-Gal@ZIF-8-2, **(d)** GOx/HRP/β-Gal@ZIF-8-2, **(e)** HRP/β-Gal/GOx@ZIF-8-2, **(f)** β-Gal/HRP/GOx@ZIF-8-2, **(g)** GOx/β-Gal/HRP@ZIF-8-2, The scale bar is 1 µm.


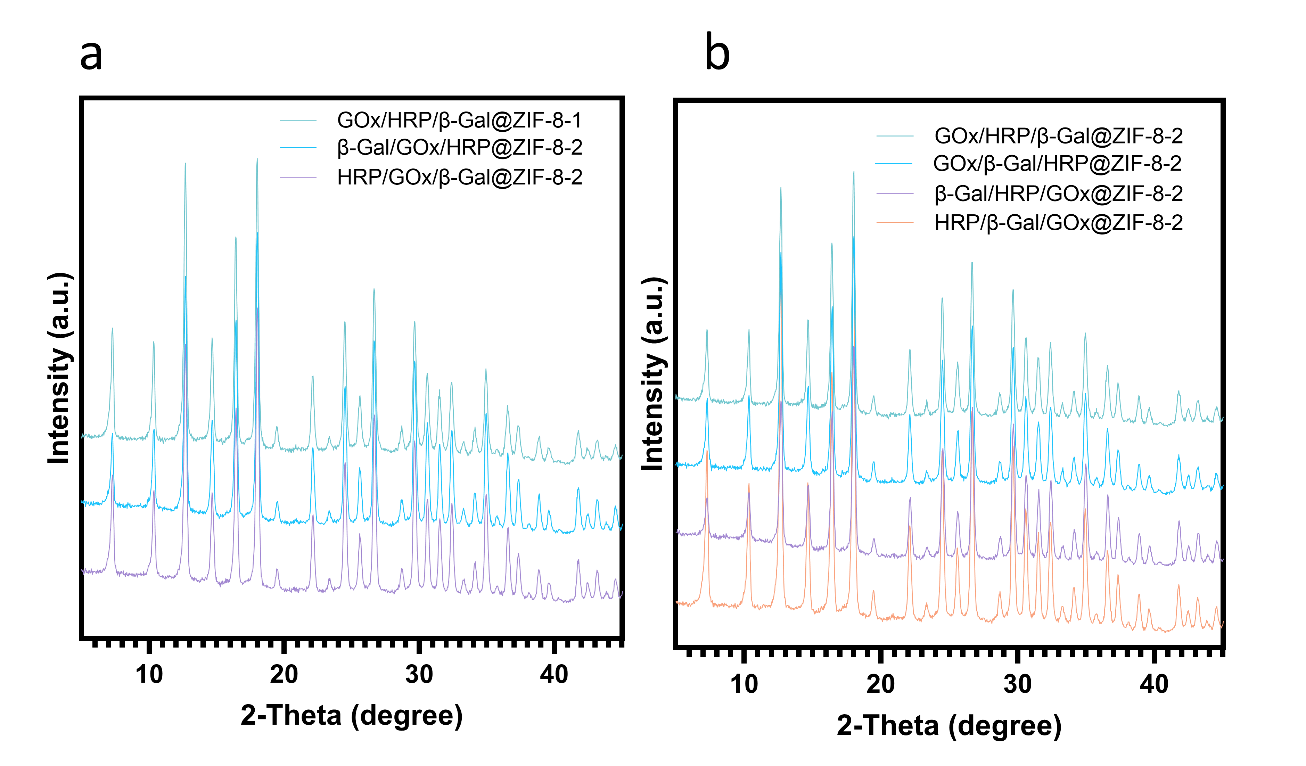


**Figure S28.** XRD patterns. (a) GOx/β-Gal/HRP@ZIF-8-1 β-Gal/GOx/HRP@ZIF-8-2, and HRP/GOx/β-Gal@ZIF-8-2. (b) GOx/HRP/β-Gal@ZIF-8-2, HRP/β-Gal/GOx@ZIF-8-2, β-Gal/HRP/GOx@ZIF-8-2, GOX/β-Gal/HRP@ZIF-8-2_._


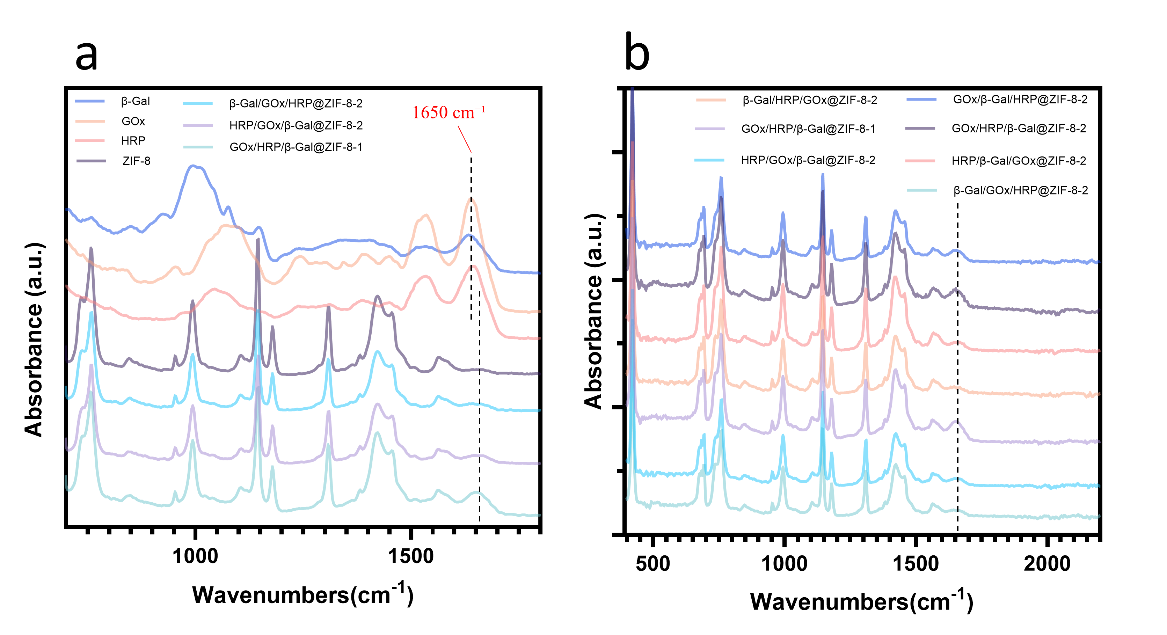


**Figure S29.** The FTIR spectra of GOx/HRP/β-Gal samples. The full spectra with dashed lines marking the amide I band.

**Figure S30.** The THz-Far-IR spectra of GOx/β-Gal/HRP@ZIF-8-1, β-Gal/GOx/HRP@ZIF-8-2, HRP/GOx/β-Gal@ZIF-8-2, GOx/HRP/β-Gal@ZIF-8-2, HRP/β-Gal/GOx@ZIF-8-2, β-Gal/HRP/GOx@ZIF-8-2, GOx/β-Gal/HRP@ZIF-8-2, β-Gal/GOx/HRP@ZIF-8-3.


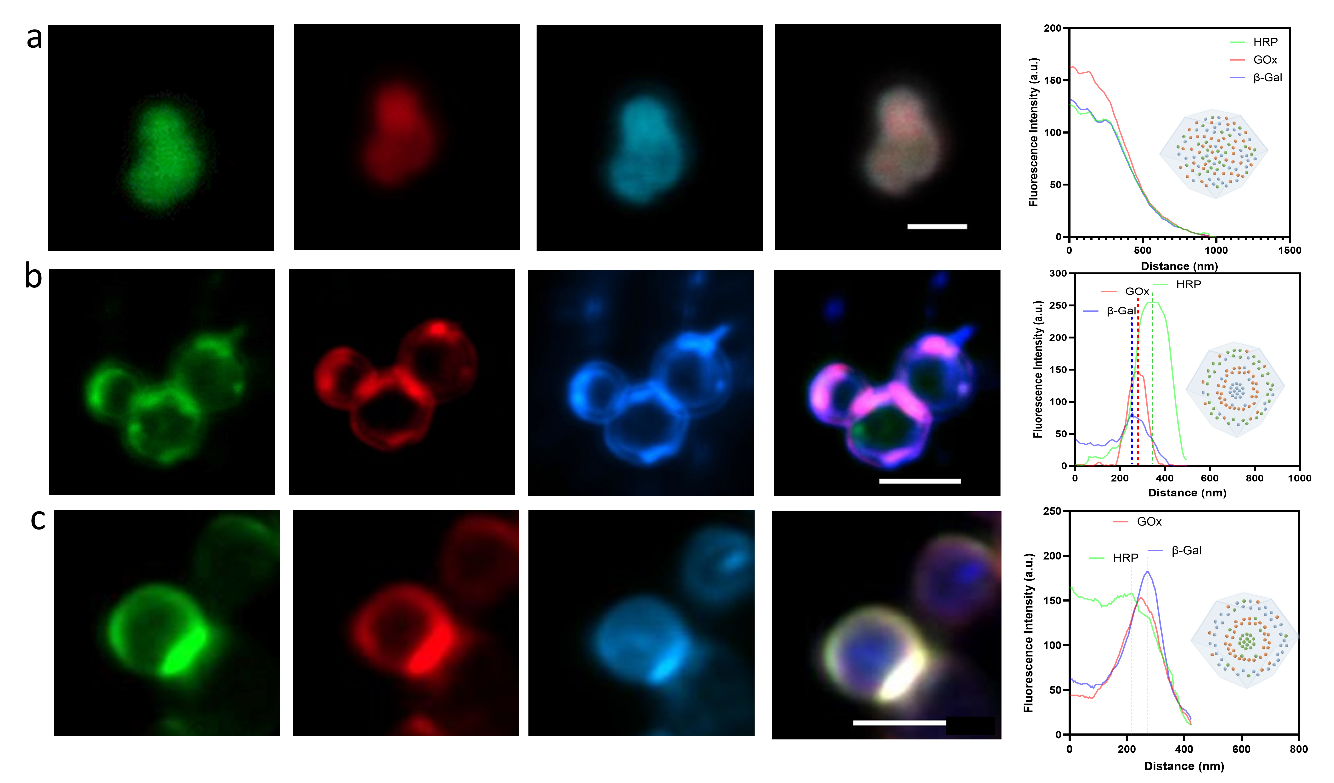


**Figure S31.** The CLSM images display the following samples: (a) β-Gal/GOx/HRP@ZIF-8-1, (b) HRP/GOx/β-Gal@ZIF-8-2, (c) β-Gal/GOx/HRP@ZIF-8-2. The scale bar is 1 μm. The green, red, and blue channels represent β-Gal, GOx, and HRP fluorescence, respectively. The right image in each row shows a fluorescence intensity profile analyzed by ImageJ, and the schematic images.


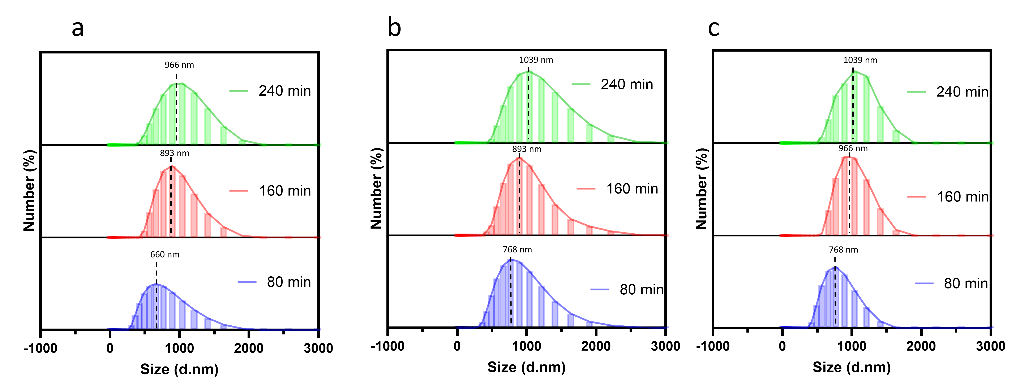


**Figure S32.** Particle size analysis of β-Gal/GOx/HRP@ZIF-8-1 (a), β-Gal/GOx/HRP@ZIF-8-2 (b), and β-Gal/GOx/HRP@ZIF-8-3 (c) via DLS.


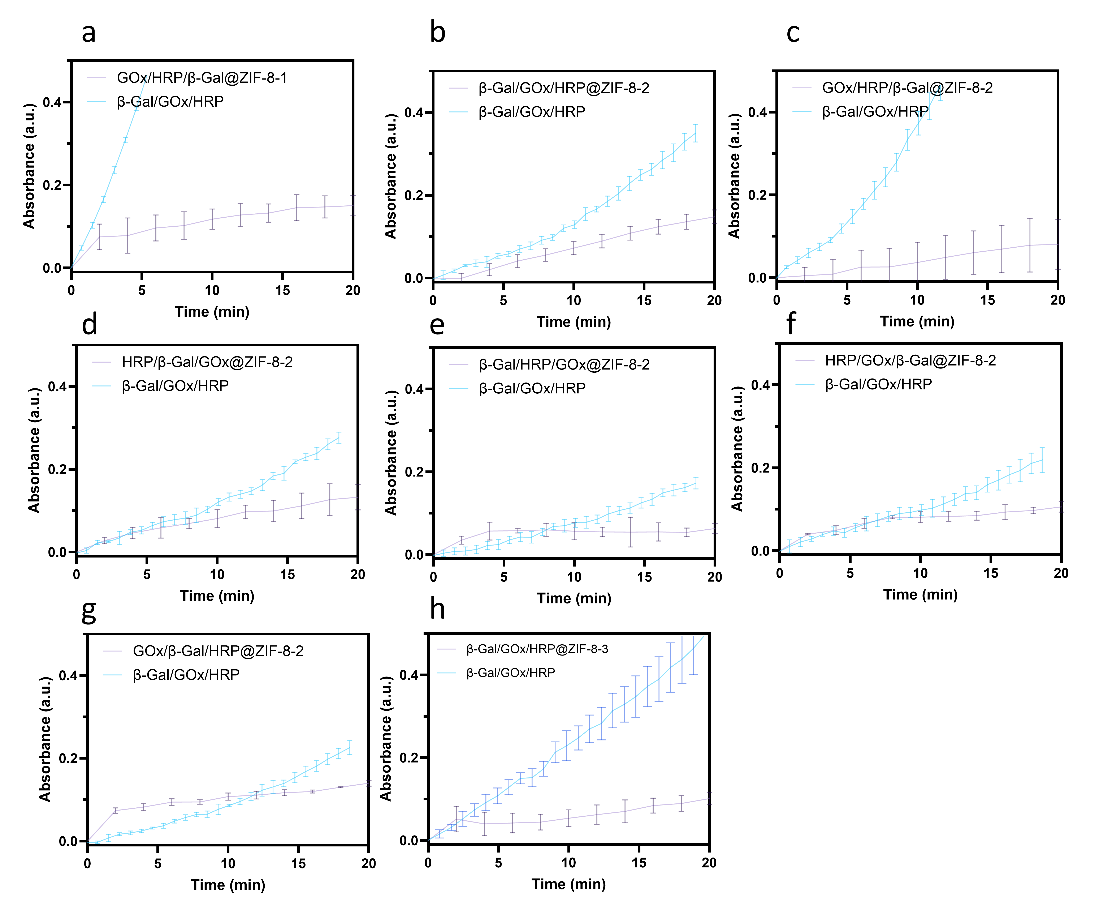


**Figure S33.** The comparison of enzymatic activity of three-enzyme@MOF and their free counterparts. (a) β-Gal/GOx/HRP@ZIF-8-1, (b) β-Gal/GOx/HRP@ZIF-8-2, (c) GOx/HRP/β-Gal@ZIF-8-2, (d) HRP/β-Gal/GOx@ZIF-8-2, (e) β-Gal/HRP/GOx@ZIF-8-2, (f) HRP/GOx/β-Gal@ZIF-8-2, (g) GOx/β-Gal/HRP@ZIF-8-2, (h) β-Gal/GOx/HRP@ZIF-8-3. The same amount of free enzymes was used for comparison based on the loading efficiency.

**Fig S34.** The reusability test of β-Gal/GOx/HRP@ZIF-8-1 in five consecutive cycles.
